# A dual-response BODIPY-based fluorescent probe for the discrimination of glutathione from cystein and homocystein†
†Electronic supplementary information (ESI) available: Synthesis, spectroscopic properties, NMR and mass spectra. See DOI: 10.1039/c5sc00216h



**DOI:** 10.1039/c5sc00216h

**Published:** 2015-02-18

**Authors:** Feiyi Wang, Li Zhou, Chunchang Zhao, Rui Wang, Qiang Fei, Sihang Luo, Zhiqian Guo, He Tian, Wei-Hong Zhu

**Affiliations:** a Key Laboratory for Advanced Materials and Institute of Fine Chemicals , Shanghai Key Laboratory of Functional Materials Chemistry , Collaborative Innovation Center for Coal Based Energy (i-CCE) , East China University of Science & Technology , Shanghai 200237 , P. R. China . Email: zhaocchang@ecust.edu.cn ; Email: whzhu@ecust.edu.cn; b Shanghai Key Laboratory of New Drug Design , School of Pharmacy , East China University of Science & Technology , Shanghai 200237 , P. R. China

## Abstract

*In situ* monitoring of intracellular thiol activity in cell growth and function is highly desirable. However, the discriminative detection of glutathione (GSH) from cysteine (Cys) and homocystein (Hcy) and from common amino acids still remains a challenge due to the similar reactivity of the thiol groups in these amino acids. Here we report a novel strategy for selectively sensing GSH by a dual-response mechanism. Integrating two independent reaction sites with a disulfide linker and a thioether function into a fluorescent BODIPY-based chemsensor can guarantee the synergetic dual-response in an elegant fashion to address the discrimination of GSH. In the first synergetic reaction process, the thiol group in GSH, Cys and Hcy induces disulfide cleavage and subsequent intramolecular cyclization to release the unmasked phenol-based BODIPY (*discriminating thiol amino acids from other amino acids*). In the second synergetic process, upon the substitution of the thioether with the nucleophilic thiolate to form a sulfenyl-BODIPY, only the amino groups of Cys and Hcy, but not that of GSH, undergo a further intramolecular displacement to yield an amino-substituted BODIPY. In this way, we make full use of the kinetically favorable cyclic transition state in the intramolecular rearrangement, and enable photophysical distinction between sulfenyl- and amino-substituted BODIPY for allowing *the discriminative detection of GSH over Cys and Hcy* and *thiol-lacking amino acids* under physiological conditions. Moreover, this probe exhibits a distinguishable ratiometric fluorescence pattern generated from the orange imaging channel to the red channel, which proves the differentiation of GSH from Cys and Hcy in living cells.

## Abstract 2

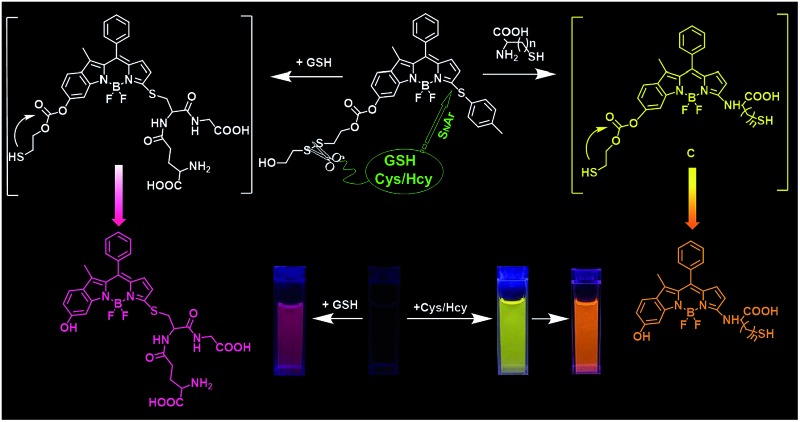
By employing a dual response approach, distinguishable fluorescence signals are initiated by GSH-mediated and Cys/Hcy-induced cascade reactions, thus allowing selective detection.

## Introduction

Since intracellular thiols, such as glutathione (GSH), cysteine (Cys) and homocystein (Hcy), play pivotal roles in physiological and pathological events, it is highly desirable to develop sensing probes to monitor and quantify *in situ* the activity of these thiols in cell growth and function.^1^ Specifically, GSH is the most abundant intracellular thiol with a concentration in the millimolar range,^2^ functioning as an essential endogenous antioxidant primarily involved in maintaining biological redox homeostasis.^3^ An inappropriate level of GSH is directly associated with cancer, Alzheimer's disease and other ailments. Accordingly, the dynamics and the quantification of GSH has become an object of great interest in the sensing community.

**Scheme 1:**
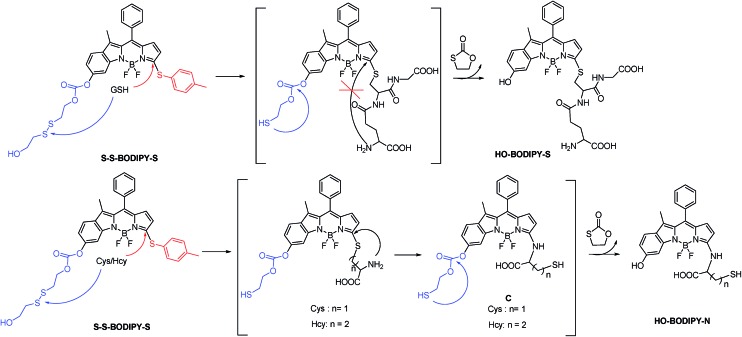
Chemical structure of **S–S-BODIPY-S** and the discriminative sensing mechanisms of **S–S-BODIPY-S** toward GSH, Cys and Hcy. Note: the intramolecular rearrangement by a five- or six-membered cyclic transition state with Cys or Hcy to form the amino-BODIPY is kinetically favored. In contrast, the bulkiness of GSH would significantly hinder the intramolecular rearrangement, thus offering the sulfenyl-BODIPY (**HO-BODIPY-S**).

To date, significant progress has been achieved in the development of fluorescent probes^4^ toward the detection of thiols by exploiting the strong nucleophilicity of the thiol group.^5–9^ However, the discriminative detection of GSH from Cys and Hcy still remains a tough task.^10^ This challenge arises from the similar reactivity of the thiol groups in these amino acids. To achieve differentiating detection, we focus on a dual-response fluorescent chemosensor; that is, the specific incorporation of two independent reaction sites with synergetic response toward these thiol-containing amino acids. Herein we present the synthesis and biological evaluations of a BODIPY-based probe, **S–S-BODIPY-S**, consisting of two key independent reaction sites with a disulfide linker and a thioether function (Scheme 1). As demonstrated, in the first synergetic reaction process for discriminating thiol amino acids (GSH, Cys and Hcy) over other amino acids, the disulfide (S–S) bond could be cleaved by the thiol group, followed by intramolecular cyclization and cleavage of a neighboring carbonate bond, thus triggering the unmasking of the hydroxyl group to afford the phenol-based BODIPY. In the second synergetic step for discriminating GSH over Cys and Hcy, upon the substitution of the thioether with the nucleophilic thiolate to form a sulfenyl-BODIPY, the subsequent intramolecular displacement takes place driven only by the amino groups of Cys or Hcy but not of GSH, yielding an amino-substituted BODIPY (Scheme 1). As a consequence, GSH triggers the transformation from **S–S-BODIPY-S** to **HO-BODIPY-S**, while Cys and Hcy produce **HO-BODIPY-N**. It should be noted that, in the second synergetic step, the kinetically favorable cyclic transition state is critical for discriminating GSH over Cys and Hcy. To be precise, the bulkiness of GSH significantly hinders the intramolecular rearrangement, thus offering only the production of sulfenyl-BODIPY. Given the remarkably different photophysical properties of sulfenyl- and amino-substituted BODIPY, the dual-response probe provides an easily distinguishable fluorescence signal to distinguish GSH from Cys and Hcy. To the best of our knowledge, this is the first fluorescent chemosensor that directly explores two synergetic reaction sites to achieve the selective determination of GSH from Cys and Hcy with a ratiometric bioimaging mode in living cells.

## Results and discussion

**Scheme 2:**
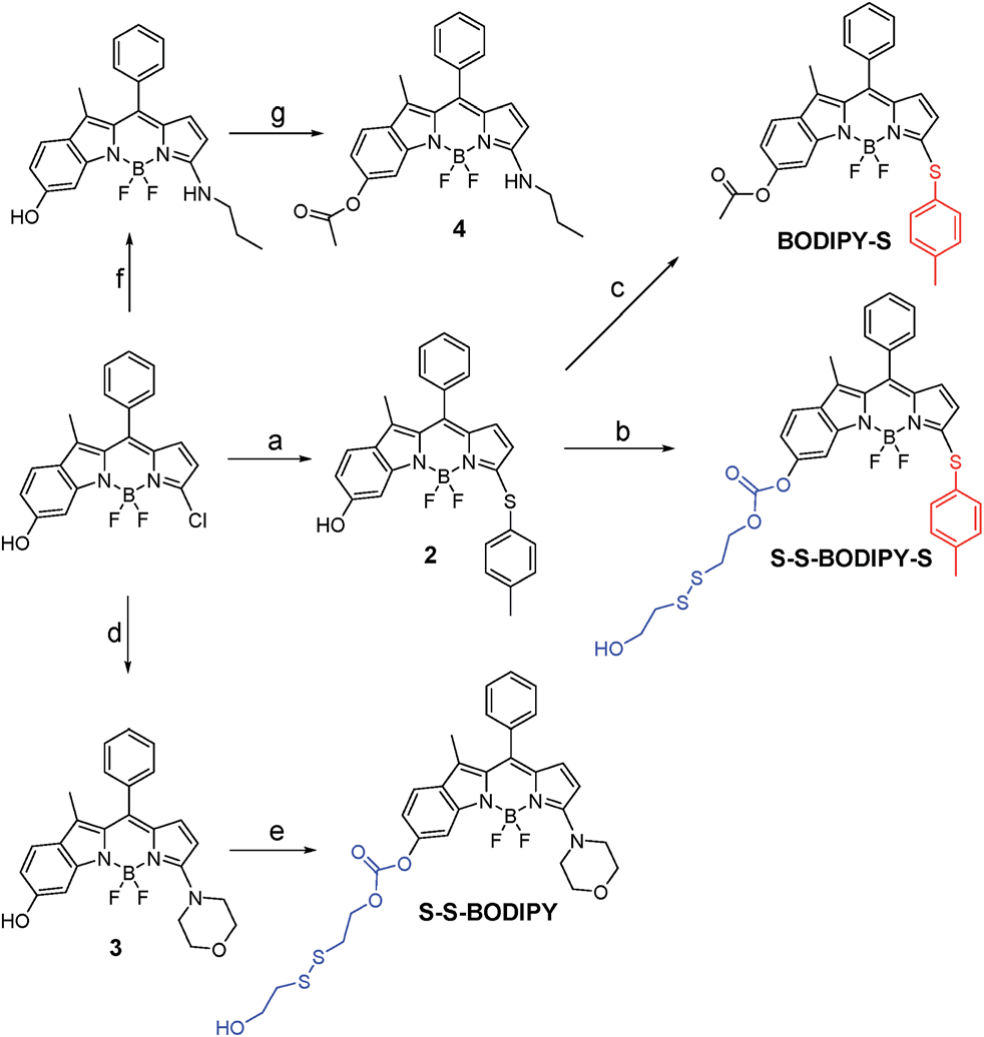
Synthesis of **S–S-BODIPY-S** and two model compounds **BODIPY-S** and **S–S-BODIPY**. Reaction conditions: (a) *p*-thiocresol, Et_3_N and CH_3_CN, rt, 88%; (b) 1,2-dichloroethane, 1,1-carbonyldiimidazole and bis(2-hydroxyethyl)disulfide, reflux, 14%; (c) DMAP, DCM and acetic anhydride, rt, 83%; (d) morpholine and CH_3_CN, rt, 90%; (e) 1,2-dichloroethane, 1,1-carbonyldiimidazole and bis(2-hydroxyethyl)disulfide, reflux, 20%; (f) propylamine and CH_3_CN, rt, 90%; (g) Et_3_N, DCM and acetic anhydride, rt, 85%.

As is well known, the spectroscopic properties of BODIPY are very sensitive to substitution on the dipyrromethene core.^10–12^ For instance, sulfenyl- and amino-substituted BODIPYs display distinct photophysical properties in absorption and emission spectra. With this in mind, we designed and evaluated a dual-response probe, **S–S-BODIPY-S**. Its two model compounds (**BODIPY-S** and **S–S-BODIPY**) were also synthesized as outlined in Scheme 2. **HO-BODIPY-Cl** smoothly reacted with *p*-thiocresol or morpholine through a nucleophilic aromatic substitution to offer **2** and **3**. The hydroxyl group was then activated by *N*,*N*′-carbonyldiimidazole (CDI) and coupled with 2,2′-dithiodiethanol to form **S–S-BODIPY-S** in 14% yield.

Initially, we determined the reactivity of the disulfide and thioether with the common thiol-containing and thiol-lacking amino acids using the two model compounds **S–S-BODIPY** and **BODIPY-S**. **BODIPY-S** displayed an absorption band at 556 nm and was almost non-fluorescent. Upon addition of GSH and other thiol-lacking amino acids to a solution of **BODIPY-S** in an acetonitrile/HEPES buffer (1 : 1, v/v, 20 mM, pH 7.4, Fig. S1 in ESI†), minimal changes in the absorption and fluorescence spectra were observed. However, a mass study manifested a peak at 718.1 (corresponding to [**Ac-BODIPY-S** + Na]^+^), showing that the substitution of thioether by GSH proceeded readily (Fig. S2 and Scheme S1†). In the case of Cys and Hcy, a rapid decrease in the absorption band of free **BODIPY-S** at 556 nm was observed, along with the simultaneous buildup of a new band at 488 nm. Additionally, a remarkable 145-fold increase in the fluorescence intensity at 552 nm was found (Fig. S1†). Mass studies with a peak at 508.1 (identical to [**Ac-BODIPY-N** – H]^–^) indicated that the thioether unit in **BODIPY-S** was replaced by Cys (Fig. S2†). The observed photophysical properties matched well with the amino-BODIPY **4** (Fig. S3†), indicative that the new product was an amino-BODIPY. Based on the reaction products and previous works,^10^ we can reason that the sulfenyl-substituted BODIPY is initially formed through nucleophilic substitution, followed by an intramolecular attack at the thioether carbon by the amino group in Cys or Hcy through a five- or six-membered transition state, yielding the amino-substituted BODIPY (Scheme S1†). This cascade substitution-intramolecular displacement mechanism is further supported by the fact that **BODIPY-S** displayed no response to the thiol-lacking amino acids, suggestive of no direct amino-induced substitution reaction. In the case of GSH, an unfavorable macrocyclic transition state can hinder the formation of amino-BODIPY. That is, the bulkiness of GSH significantly hinders the intramolecular rearrangement, thus offering only the sulfenyl-BODIPY. In this regard, the discriminative detection of Cys/Hcy over GSH and thiol-lacking amino acids might be expected by **BODIPY-S**. However, we cannot distinguish GSH and thiol-lacking amino acids with **BODIPY-S** because the substitution of thioether by GSH induces a minimal fluorescence change, which is very similar to the thiol-lacking amino acids.

The response behavior of model compound **S–S-BODIPY** toward the common thiol-containing and thiol-lacking amino acids was further determined. It was found that only the three thiol-containing amino acids (GSH, Cys and Hcy) elicited a time-dependent ratiometric fluorescence change, resulting in a decrease in the emission band at 563 nm and a concomitant increase of a new band at 598 nm (Fig. S4†). The TLC, ^1^H NMR and HRMS experiments all showed that the disulfide bond in **S–S-BODIPY** was cleaved by the specific nucleophilic thiol group, followed by an intramolecular cyclization to break down the carbonate bonds and yield compound **3** (Scheme S2 and Fig. S5†). Clearly, **S–S-BODIPY** is susceptible to discriminating thiol-containing amino acids from other amino acids since the disulfide bond is inactive toward thiol-lacking amino acids. Nevertheless, we cannot expect the disulfide reduction mediated by the thiol group to discriminate between thiol-containing amino acids such as GSH, Cys and Hcy.

**Fig. 1:**
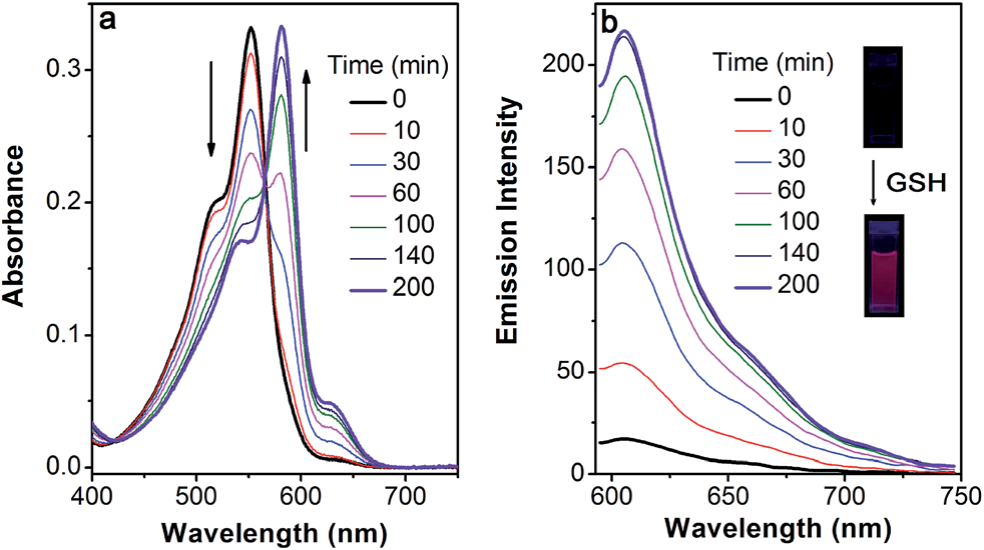
Time-dependent spectral changes of **S–S-BODIPY-S** (5 μM) in the absence and presence of 5 mM GSH in an acetonitrile/HEPES buffer (1 : 1, v/v, 20 mM, pH 7.4) at 37 °C. (a) Absorption and (b) emission spectra, *λ*_ex_ = 565 nm. Inset: the fluorescence color change of **S–S-BODIPY-S** in the presence of GSH.

Based on the above-mentioned essential weakness of the two model systems, the spectral properties of **S–S-BODIPY-S** possessing the two key independent reaction sites of a disulfide linker and a thioether function were further investigated. We envisioned that the synergetic dual-responses of the two reaction sites can guarantee the selective detection of GSH. Initially, **S–S-BODIPY-S** exhibited a strong absorption band at 552 nm as well as a weak emission peak at 605 nm with a quantum yield of around 0.001. As shown in Fig. 1, addition of 5 mM GSH to a solution of **S–S-BODIPY-S** in an acetonitrile/HEPES buffer (1 : 1, v/v, 20 mM, pH 7.4) triggered a dramatic decrease in the absorption band at 552 nm, along with a simultaneous absorption buildup at 581 nm, displaying a distinct isosbestic point at 565 nm. In the fluorescence spectra, addition of GSH elicited a red fluorescence turn-on signal at 605 nm, enabling **S–S-BODIPY-S** to be a promising probe for GSH. The increment of fluorescence intensity is time-dependent and the observed rate constant was determined to be 0.0175 min^–1^ under pseudo-first-order conditions (Fig. S6†). It has been demonstrated that GSH can displace the thioether in **BODIPY-S** and unmask the protective ester group in **S–S-BODIPY**. Therefore, we reasoned that the incubation of **S–S-BODIPY-S** with GSH triggered both reactions to give **HO-BODIPY-S** with a quantum yield of around 0.06, as illustrated in Scheme 1. The HRMS study also revealed the cleavage of the disulfide and thioether linkers to form **HO-BODIPY-S** (Fig. S7†). The reaction product manifests a mass peak at 654.1993, identical to [**HO-BODIPY-S**]^+^.

**Fig. 2:**
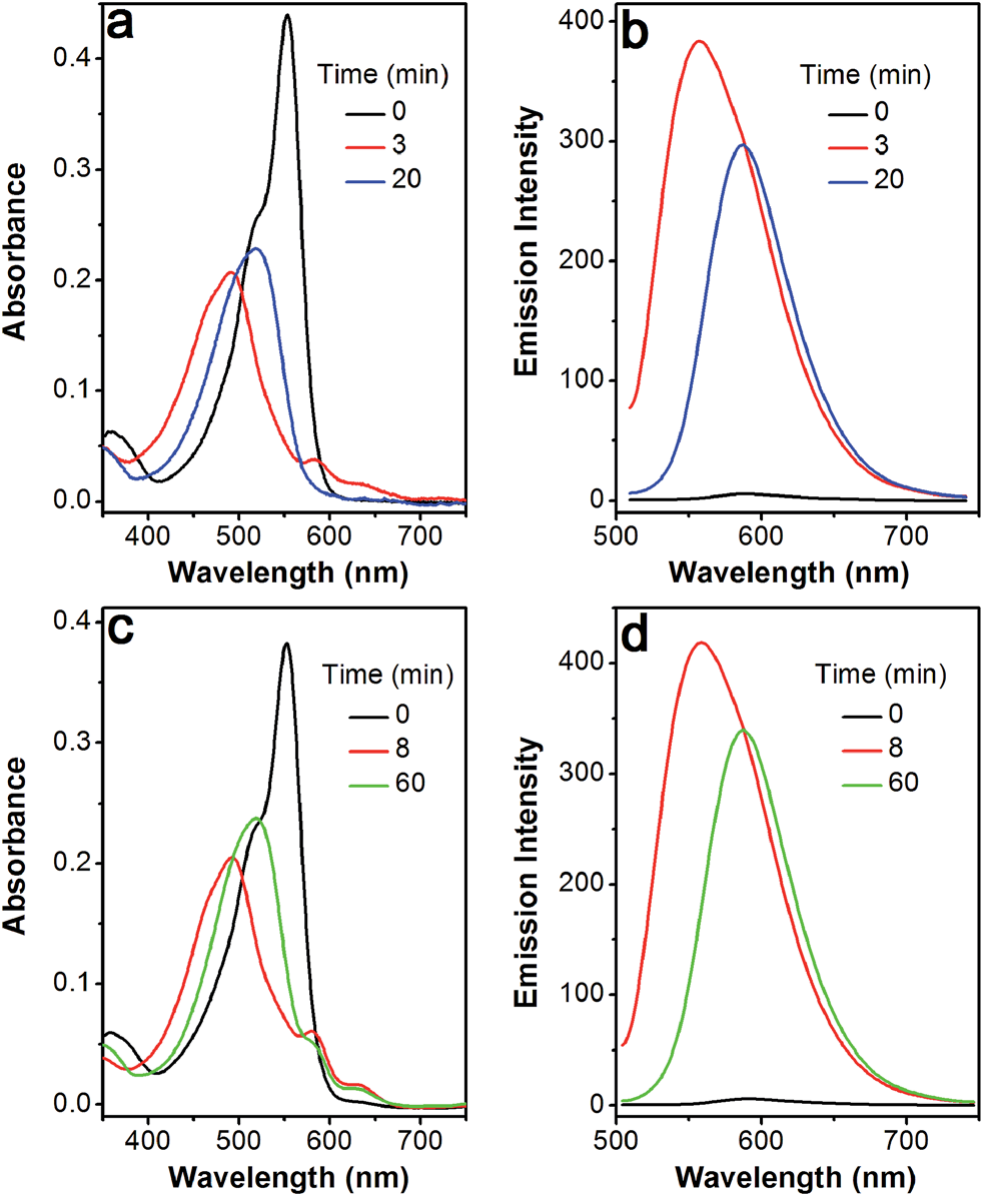
Time-dependent spectral changes of **S–S-BODIPY-S** (5 μM) in the absence and presence of Cys (5 mM) or Hcy (5 mM) in an acetonitrile/HEPES buffer (1 : 1, v/v, 20 mM, pH 7.4) at 37 °C. Cys: (a) absorption and (b) emission spectra; Hcy: (c) absorption and (d) emission spectra. *λ*_ex_ = 490 nm.

The profile changes of the spectra were then investigated in the presence of Cys or Hcy (Fig. 2 and S8†). A biphasic nature in the changes of the spectra was observed upon the addition of Cys. In the initial 3 min, the original absorption at 552 nm became diminished and a new absorption band at 490 nm increased simultaneously. Subsequently, the absorption band at 490 nm shifted to 520 nm. The optical density value reached a plateau within 20 min. Additionally, a remarkable increase in fluorescence intensity at 556 nm was observed upon excitation at 490 nm during the initial 3 min. A fluorescence red-shift then took place, and finally shifted to 587 nm. The easy-to-monitor fluorescence color change was observed from dark to yellow and eventually to orange (Fig. S9†). Based on the two model compounds, we can infer the formation of **HO-BODIPY-N** with a quantum yield of around 0.13. Again, the HRMS study also revealed the release of **HO-BODIPY-N** by breaking of the disulfide and thioether bonds (Fig. S7†). Similar spectroscopic changes were also found in the presence of Hcy, with the exception of a prolonged incubation time. The spectral profile variations were presumably due to the formation of intermediate **C** (Scheme 1) with a yellow fluorescence color (556 nm). Since the subsequent intramolecular cyclization and unmasking of the hydroxyl group introduced the removal of the electron-withdrawing ester, **HO-BODIPY-N** exhibited a red-shifted fluorescence at 587 nm.

Taken together, GSH triggered the transformation from **S–S-BODIPY-S** to **HO-BODIPY-S**, while Cys and Hcy led to **HO-BODIPY-N** (Scheme 1). Thus, the photophysical features of **S–S-BODIPY-S** in the presence of GSH are significantly different from those in the presence of Cys or Hcy, enabling **S–S-BODIPY-S** to selectively detect GSH over Cys and Hcy under physiological conditions. As a consequence, the incorporation of the two independent disulfide linker and thioether functions realizes the synergetic response to GSH, Cys and Hcy. In the first synergetic step, the thiol group in the three thiol amino acids (GSH, Cys and Hcy) induces disulfide cleavage and subsequent intramolecular cyclization to release the unmasked phenol-based BODIPY (*discriminating thiol amino acids from other amino acids*). In the second synergetic step, after the substitution of the thioether with the nucleophilic thiolate to form the sulfenyl-BODIPY, only the amino groups of Cys and Hcy but not that of GSH undergo further intramolecular displacement to yield an amino-substituted BODIPY (*discriminating GSH over Cys and Hcy in thiol amino acids*).

**Fig. 3:**
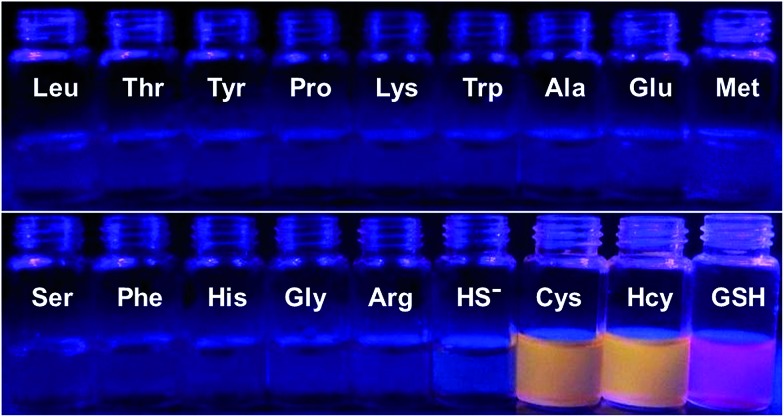
Fluorescence response of **S–S-BODIPY-S** in the presence of GSH, Cys, Hcy, HS^–^ and other related amino acids. All spectra were acquired at 2.5 h after the addition of the analytes.

To further confirm the specificity of **S–S-BODIPY-S** toward GSH, the probe was then separately incubated with various biologically relevant amino acids and hydrogen sulfide under the same physiological conditions. As can be seen from Fig. 3, **S–S-BODIPY-S** is a highly selective probe and can monitor GSH with minimum interference from other relevant analytes.

**Fig. 4:**
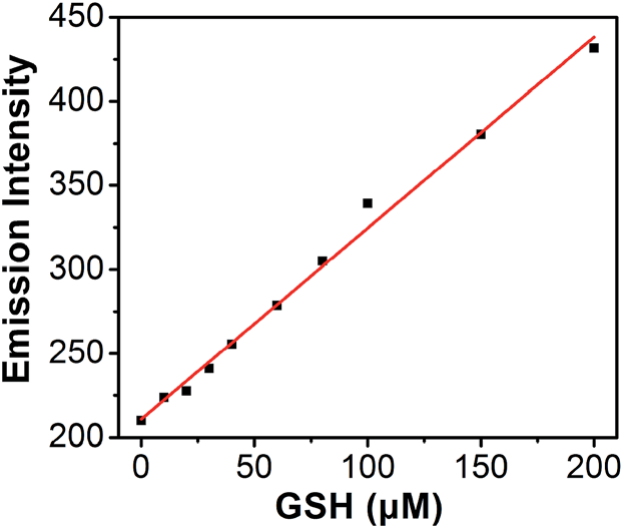
Plot of fluorescence intensity changes at 610 nm of **S–S-BODIPY-S** as a function of GSH concentration, with a detection limit of 8.5 × 10^–7^ M for GSH. Data acquired at 2.5 h after the addition of GSH.

Titration experiments were also performed to evaluate the efficiency of **S–S-BODIPY-S** in the measurement of various concentrations of GSH. It is clear that the enhancement of fluorescence intensity is GSH concentration dependent and a linear relationship can be observed with a GSH concentration of up to 200 μM (Fig. 4). The detection limit was then determined to be 8.5 × 10^–7^ M, which indicates that **S–S-BODIPY-S** can readily detect micromolar concentrations of GSH under physiological conditions.

**Fig. 5:**
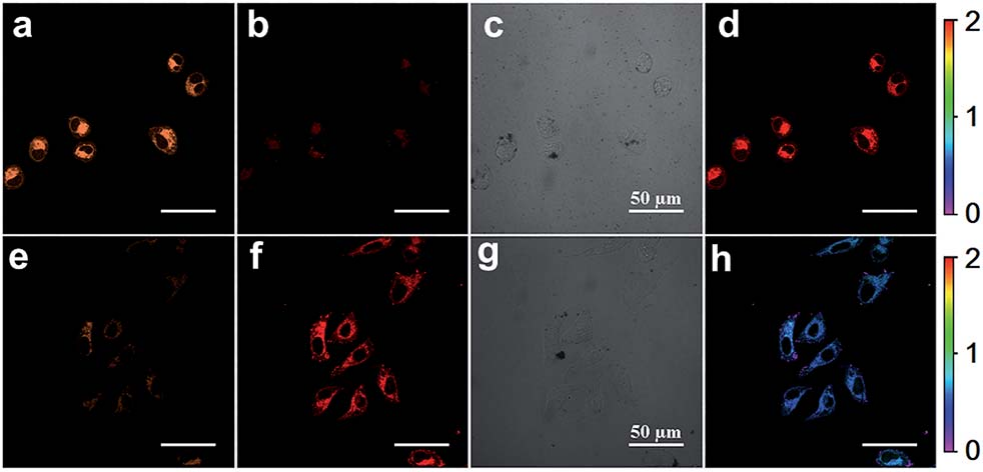
Confocal fluorescence image of (a–d) MKN-45 cells and (e–h) HeLa cells treated with **S–S-BODIPY-S** (10 μM) for 4 h; the excitation wavelength was 514 nm and the emission was collected at 600–620 nm for the red channel and 560–580 nm for the orange channel. (a and e) Fluorescence image for the orange channel; (b and f) fluorescence image for the red channel; (c and g) bright field; (d and h) ratio images generated from the orange channel to the red channel. The scale bar is 50 μm.

Inspired by the capability of **S–S-BODIPY-S** to detect GSH and Cys or Hcy with distinct fluorescence patterns, we finally sought to apply this probe in fluorescence imaging applications. Evidently, the reaction of **S–S-BODIPY-S** with GSH features a red fluorescence color, whereas that of **S–S-BODIPY-S** with Cys or Hcy gives an orange color (Fig. 3). Thus, we investigated the feasibility of the probe in detecting these thiols in a dual-color manner to interrogate the discriminative capacity of the probe. Imaging of cellular GSH in living HeLa cells was first carried out. Upon loading 10 μM of **S–S-BODIPY-S** at 37 °C, these cells displayed a bright red fluorescence image and a relatively weak orange fluorescence image (Fig. 5), showing a ratio of 0.5 from the orange channel to the red channel. In contrast, addition of *N*-methylmaleimide (a trapping reagent of thiols) to the cell culture prior to incubation with **S–S-BODIPY-S** resulted in minimal fluorescence signals in both channels (Fig. S10†). These results suggest that **S–S-BODIPY-S** is cell membrane permeable and amenable for fluorescent imaging of GSH in living cells. For the imaging of Cys and Hcy, MKN-45 cells were explored. When these cells were loaded with **S–S-BODIPY-S**, bright fluorescence signals in the orange channel were observed, whereas weak red fluorescence signals were noted. The ratio of the two emissions from the orange channel to the red channel is about 2, resulting in a remarkable 4-fold enhancement of the emission ratio compared to the image of GSH. These experiments clearly revealed that **S–S-BODIPY-S** exhibited distinguishable fluorescence images for GSH and Cys or Hcy in living cells.

## Conclusions

In conclusion, we have constructed a dual response BODIPY-based fluorescent probe for the discriminative detection of GSH from Cys and Hcy, and from common amino acids. This unique probe is designed to have two independent reaction sites: a disulfide linker and a thioether function. In the first synergetic process for discriminating thiol amino acids (GSH, Cys and Hcy) from other amino acids, the disulfide bond was cleaved by the thiol group, followed by intramolecular cyclization and cleavage of a neighboring carbonate bond, thus triggering the unmasking of the hydroxyl group to afford the phenol-based BODIPY. In the second synergetic step for discriminating GSH from Cys and Hcy, upon the substitution of thioether with the nucleophilic thiolate to form the sulfenyl-BODIPY, the subsequent intramolecular displacement takes place driven only by the amino groups of Cys or Hcy, but not by that of GSH, yielding an amino-substituted BODIPY. As a consequence, GSH triggered the production of a hydroxyl-based sulfenyl-BODIPY, while Cys or Hcy induced the formation of a hydroxyl-based amino-BODIPY. Thus, the photophysical features of **S–S-BODIPY-S** in the presence of GSH are significantly different from those in the presence of Cys and Hcy and from those of other thiol-lacking amino acids, enabling **S–S-BODIPY-S** to selectively detect GSH over Cys and Hcy under physiological conditions. Furthermore, **S–S-BODIPY-S** can monitor GSH with minimum interference from other relevant analytes. Importantly, **S–S-BODIPY-S** is cell membrane permeable and amenable for fluorescent imaging of these thiols in living cells with distinguishable fluorescence images in a dual-channel manner.
