# Supplementary material for: A dual-response BODIPY-based fluorescent probe for the discrimination of glutathione from cystein and homocystein
Source: Chem Sci. 2015 Feb 18;6(4):2584–9. doi: 10.1039/c5sc00216h (PMC5812429; doi:10.1039/c5sc00216h)

Electronic Supplementary Material (ESI) for Chemical Science.
This journal is © The Royal Society of Chemistry 2015

Electronic Supplementary Information (ESI)

A dual-response BODIPY-based fluorescent probe for the discrimination of Glutathione from Cystein and Homocystein

Feiyi Wang,[a] Li Zhou,[b] Chunchang Zhao,*[a] Rui Wang,[b] Qiang Fei,[a] Sihang Luo,[a] Zhiqian Guo,[a] He Tian,[a] Wei-Hong Zhu*[a]

[a] Key Laboratory for Advanced Materials and Institute of Fine Chemicals, Shanghai Key Laboratory of Functional Materials Chemistry, Collaborative Innovation Center for Coal Based Energy (i-CCE), East China University of Science & Technology, Shanghai 200237, P. R. China. E-mail: zhaocchang@ecust.edu.cn, whzhu@ecust.edu.cn.

[b] Shanghai Key Laboratory of New Drug Design, School of Pharmacy, East China University of Science & Technology, Shanghai 200237, P. R. China.

Table of Contents

1. Time dependent spectral changes of BODIPY-S in the presence of thiols. ……S2

2. MS of BODIPY-S with GSH, Cys and Hcy…………………..……………..…S3

3. Photophysical spectra of BODIPY 4 and BODIPY-S +Cys……………………..S4

4. Time dependent Spectral changes of S-S-BODIPY in the presence of thiols. …S5

5. NMR and MS of the product of S-S-BODIPY with thiols………..…………......S6

6. Reaction mechanism ……………………..……………………….………. S7

7. Pseudo first-order kinetic plot……….…………………………………….…...S8

8. HRMS of S-S-BODIPY-S with thiols ……………………………………..S9-S10

9. Time-dependent spectral changes of S-S-BODIPY-S with Cys/Hcy………..….S11

10. The easy-to-monitor fluorescence color change……………………………..S12

11. Fluorescent confocal image of cells. ……………………….………………..S12

12. Experimental section…….……………………………………….....….…S13-S15

13. NMR and HRMS spectra………………………..………….…..……....S16-S21

1. Time-dependent spectral changes of BODIPY-S in the presence of thiols.

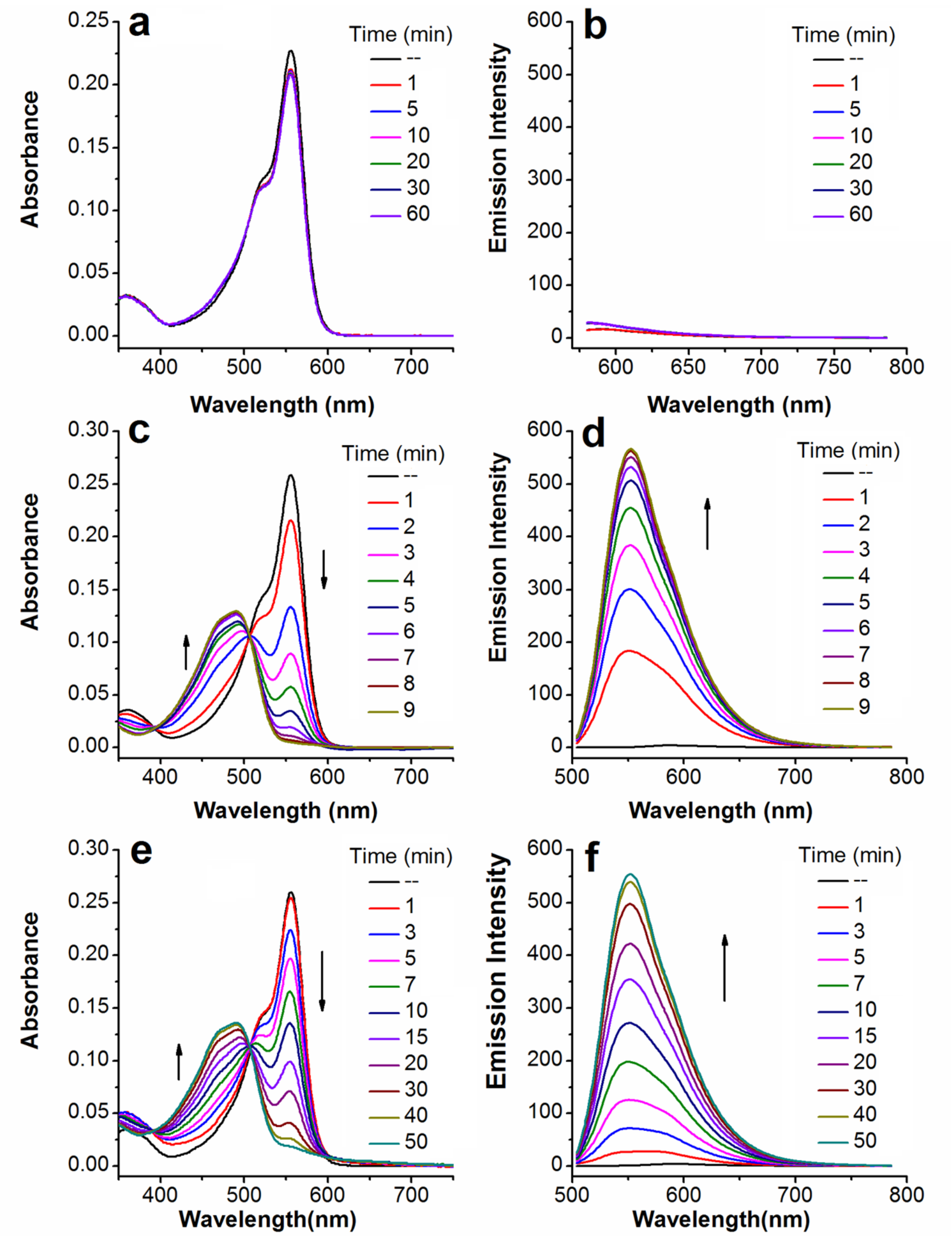

Fig. S1 Time-dependent spectral changes of **BODIPY-S** (5 μM) in the absence and presence of 5 mM thiols in acetonitrile / HEPES buffer (1:1, v/v , 20 mM, pH 7.4) at 37 °C. GSH: (a) Absorption and (b) emission spectra, λ_{ex} = 565 nm. Cys: (c) Absorption and (d) emission spectra, λ_{ex} = 480 nm. Hcy: (e) Absorption and (f) emission spectra, λ_{ex} = 480 nm.

2. MS of BODIPY-S with GSH, Cys and Hcy.

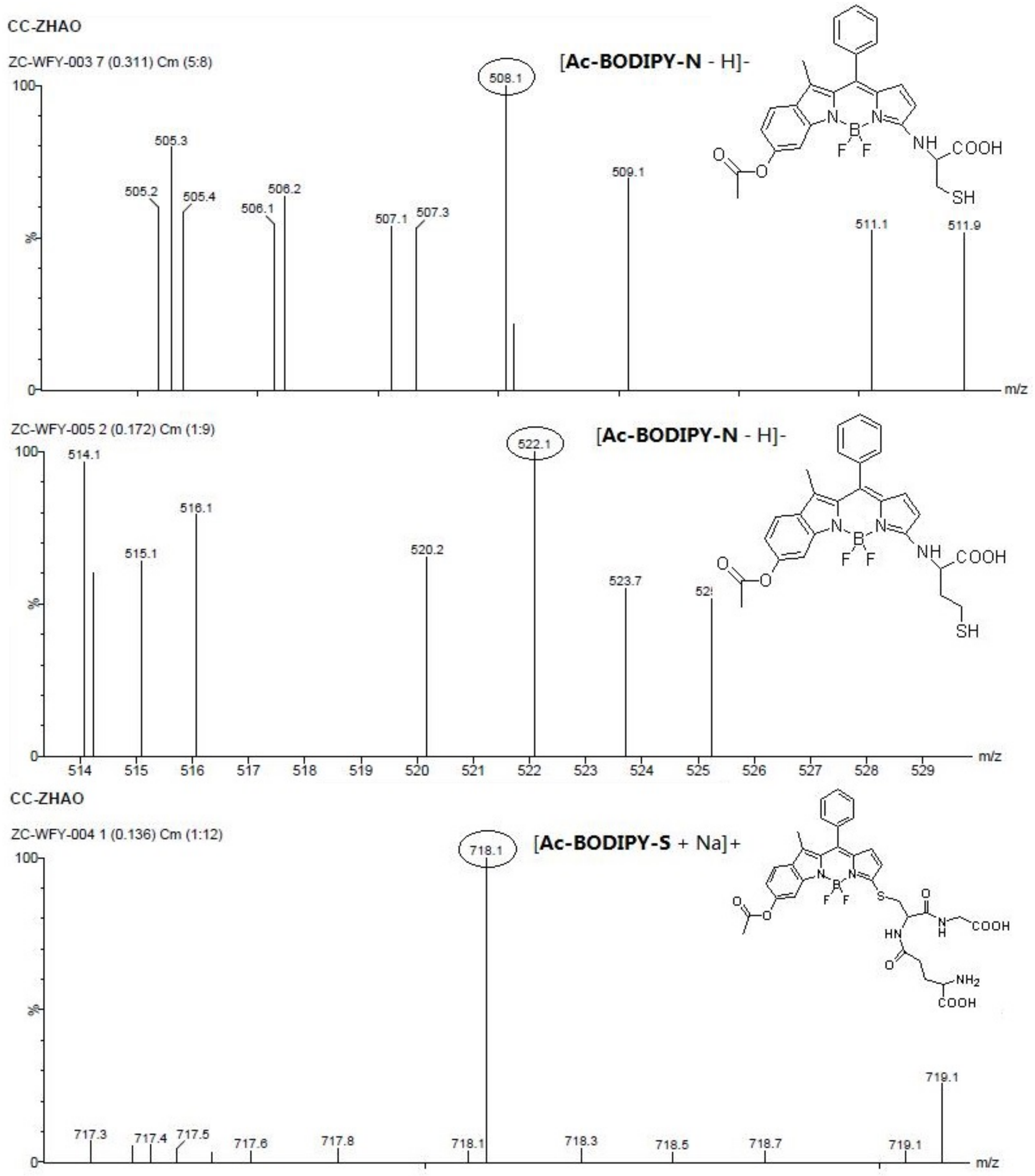

Fig. S2 MS spectra of the products from **BODIPY-S** with thiols.

3. Photophysical spectra of BODIPY 4 and BODIPY-S +Cys.

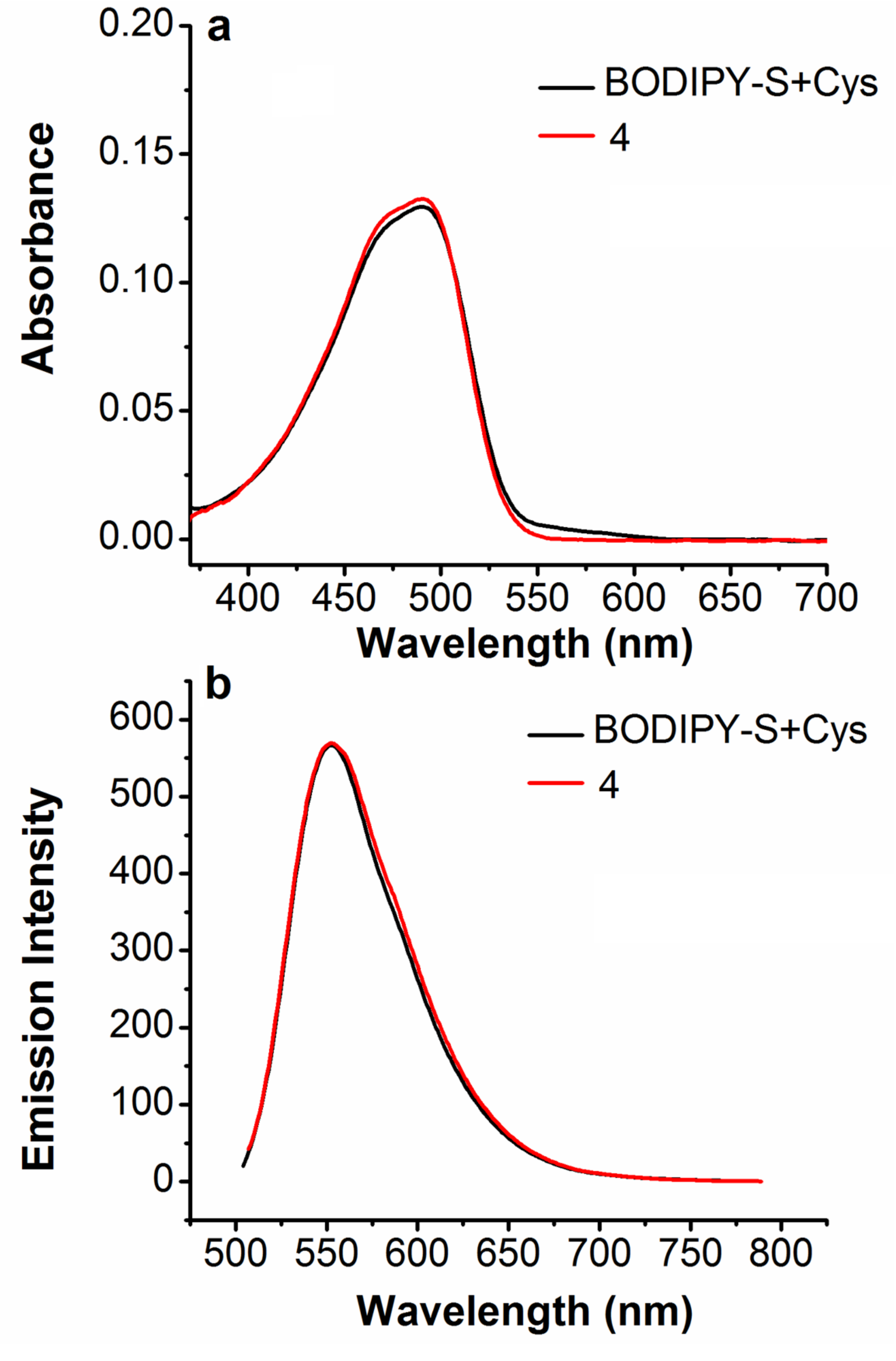

Fig. S3 Photophysical spectra of BODIPY **4** and **BODIPY-S** + Cys.

4. Time dependent spectral changes of S-S-BODIPY in the presence of thiols.

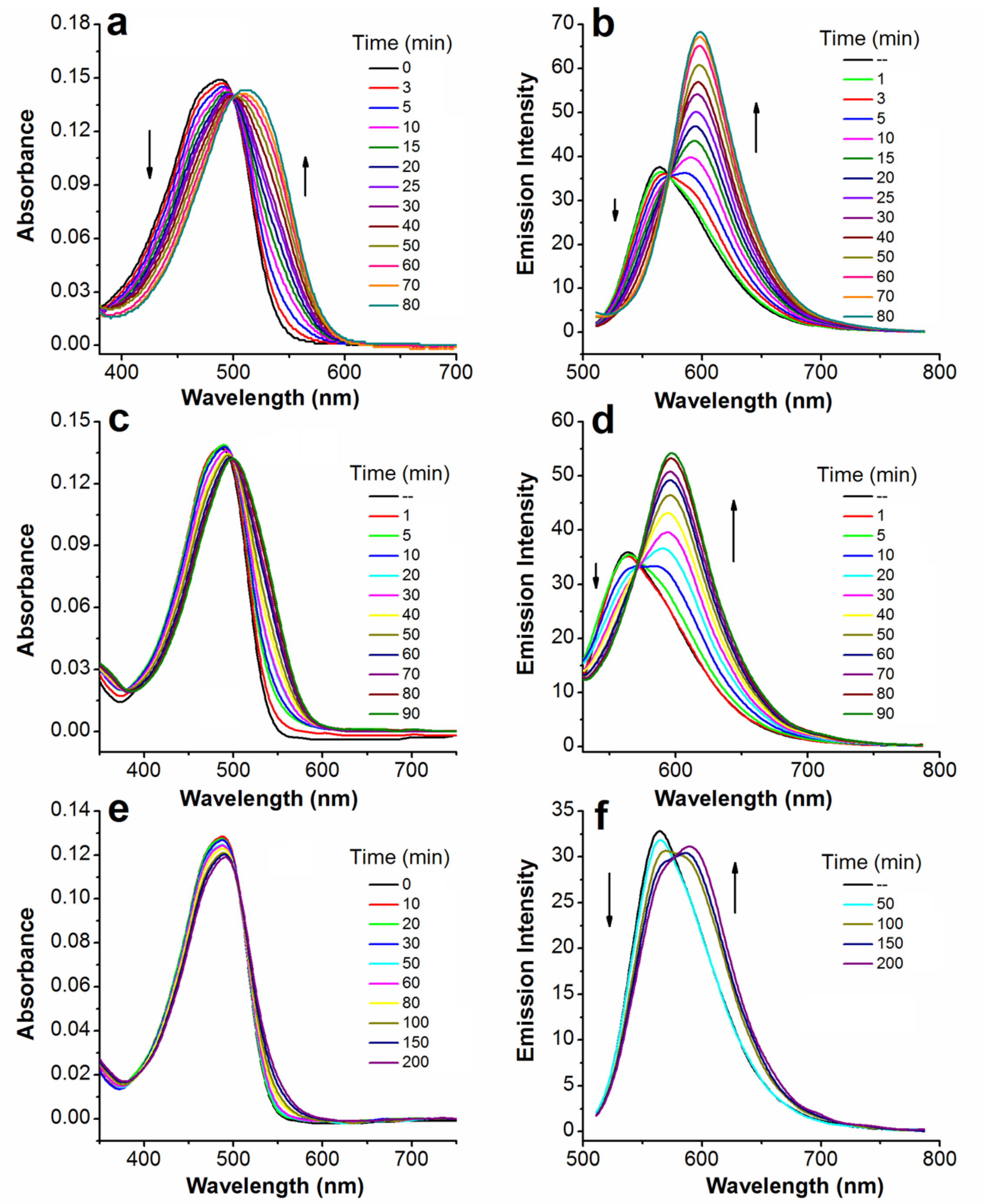

Fig. S4 Time-dependent spectral changes of **S-S-BODIPY** (5 μM) in the absence and presence of 5 mM thiols in acetonitrile / HEPES buffer (1:1, v/v, 20 mM, pH 7.4) at 37 °C. Cys: (a) Absorption and (b) emission spectra, λ_{ex} = 487 nm. Hcy: (c) Absorption and (d) emission spectra, λ_{ex} = 487 nm. GSH: (e) absorption and (f) emission spectra, λ_{ex} = 487 nm.

5. ^{1}H NMR and HRMS of the product from S-S-BODIPY with thiols.

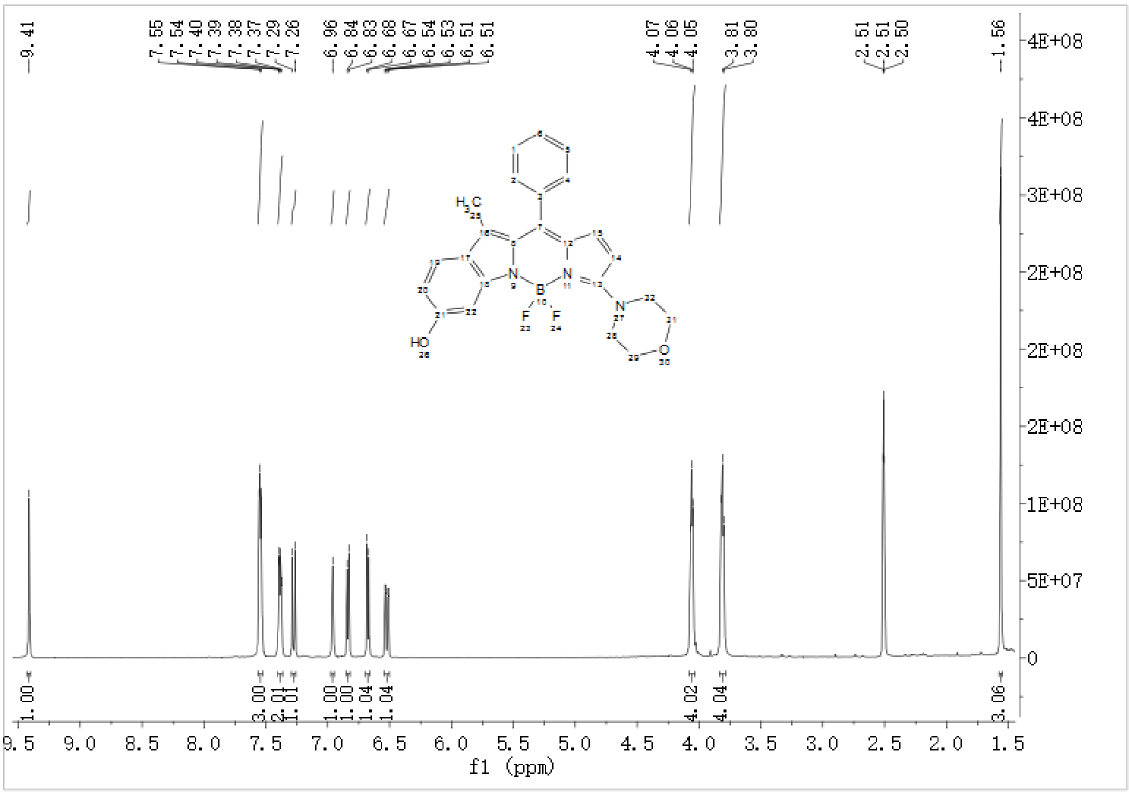

Elemental Composition Report

Single Mass Analysis
Tolerance = 30.0 mDa / DBE: min = -1.5, max = 100.0
Element prediction: Off
Number of isotope peaks used for i-FIT = 2

Monoisotopic Mass, Even Electron Ions
163 formula(e) evaluated with 21 results within limits (up to 1 best isotopic matches for each mass)
Elements Used:
C: 0-31 H: 0-33 11B: 0-1 N: 0-3 O: 0-2 F: 0-2

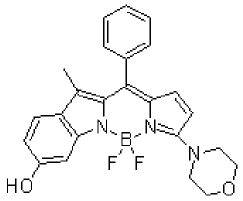

CC-ZHAO ECUST institute of Fine Chem 30-Jul-2014 20:32:49

ZC-WFY-008 36 (1.182) Cm (36:37) 1: TOF MS ES+ 1.02e+003

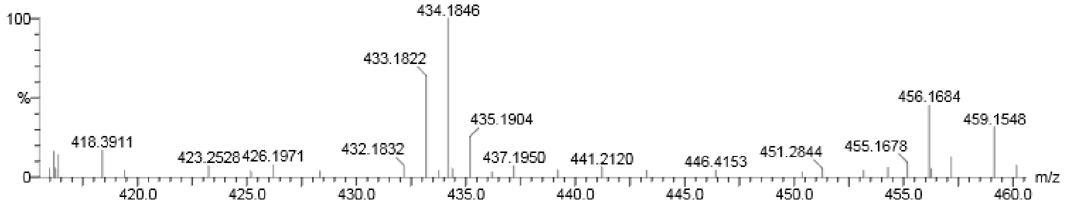

Minimum:				-1.5			
Maximum:		30.0	50.0	100.0			
Mass	Calc. Mass	mDa	PPM	DBE	i-FIT	i-FIT (Norm)	Formula
434.1846	434.1851	-0.5	-1.2	14.5	16.4	0.0	C24 H23 11B N3 O2 F2

Fig. S5 The characterization of the product from **S-S-BODIPY** with GSH, Cys or Hcy.

6. Reaction mechanism.

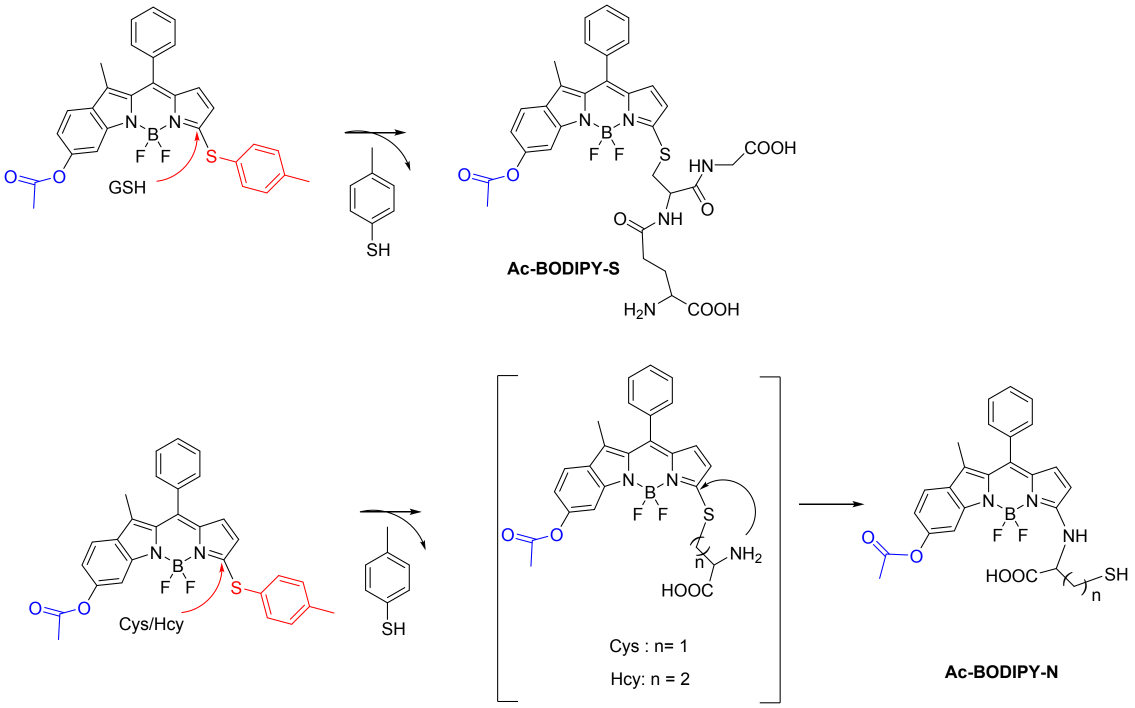

Scheme S1. Reaction mechanism of compound **BODIPY-S** toward GSH, Cys/Hcy.

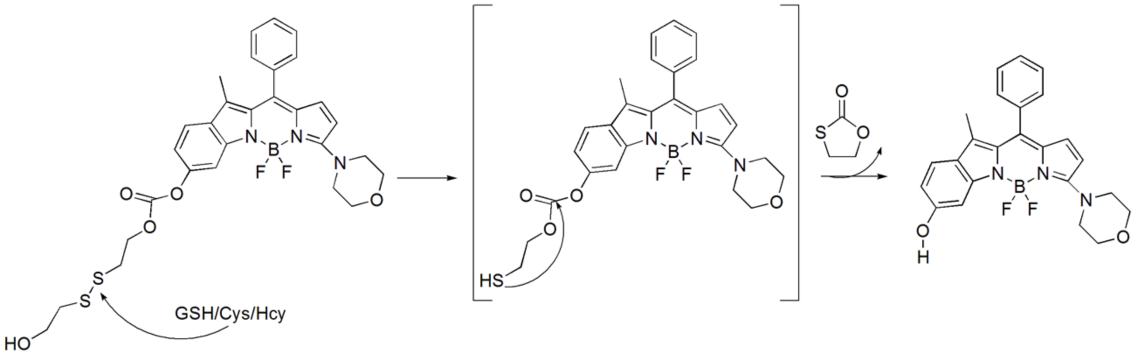

Scheme S2. Reaction mechanism of compound **S-S-BODIPY** toward GSH, Cys/Hcy.

7. Pseudo first-order kinetic plot.

Time-course kinetic measurements of **S-S-BODIPY-S** with GSH were performed using the fluorescence intensity at 605 nm. Data were collected under pseudo-first-order conditions. Spectra were acquired in acetonitrile / HEPES buffer (1:1, v/v, 20 mM, pH 7.4) at 37 °C. The pseudo-first-order rate constant for the reaction was determined by fitting the fluorescence intensity changes of the samples to the pseudo first-order equation: $\mathrm{Ln}((I_{max}-I_t)/I_{max})) = -\, k_{obs}\, t$.
Where I_t and I_{max} represent the fluorescence intensities at times t and the maximum value obtained after the reaction was complete. k_{obs} is the observed rate constant.

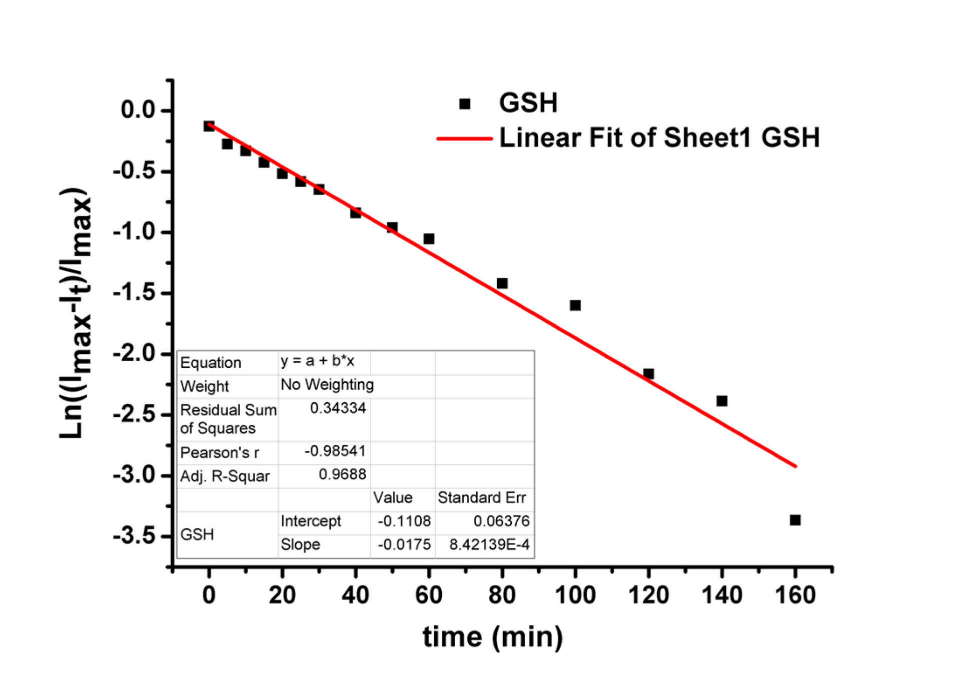

Fig. S6 Pseudo first-order kinetic plots of the reaction of **S-S-BODIPY-S** (5 μM) with GSH (5 mM).

8. HRMS of S-S-BODIPY-S with thiols.

Elemental Composition Report

Single Mass Analysis
Tolerance = 30.0 mDa / DBE: min = -1.5, max = 100.0
Element prediction: Off
Number of isotope peaks used for i-FIT = 2

Monoisotopic Mass, Even Electron Ions
556 formula(e) evaluated with 1 results within limits (up to 1 best isotopic matches for each mass)
Elements Used:
C: 0-30 H: 0-33 11B: 0-1 N: 0-5 O: 0-7 F: 0-2 S: 0-1

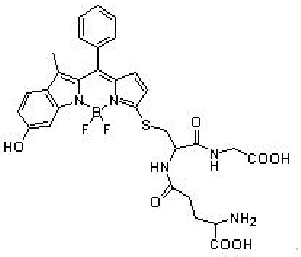

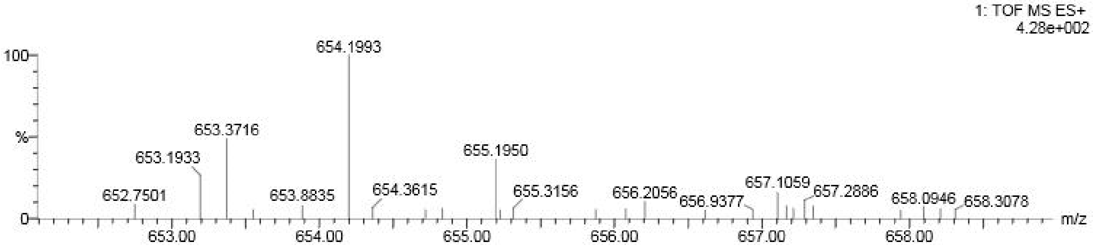

Minimum:				-1.5			
Maximum:		30.0	50.0	100.0			
Mass	Calc. Mass	mDa	PPM	DBE	i-FIT	i-FIT (Norm)	Formula
654.1993	654.2005	-1.2	-1.8	17.5	41.9	0.0	C30 H31 11B N5 O7 F2 S

Elemental Composition Report

Single Mass Analysis
Tolerance = 30.0 mDa / DBE: min = -1.5, max = 100.0
Element prediction: Off
Number of isotope peaks used for i-FIT = 2

Monoisotopic Mass, Even Electron Ions
200 formula(e) evaluated with 1 results within limits (all results (up to 1000) for each mass)
Elements Used:
C: 0-23 H: 0-150 11B: 0-1 N: 0-3 O: 0-3 F: 0-2 S: 0-1

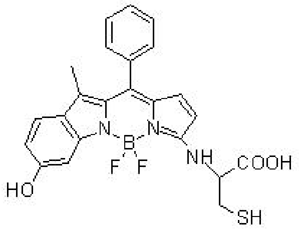

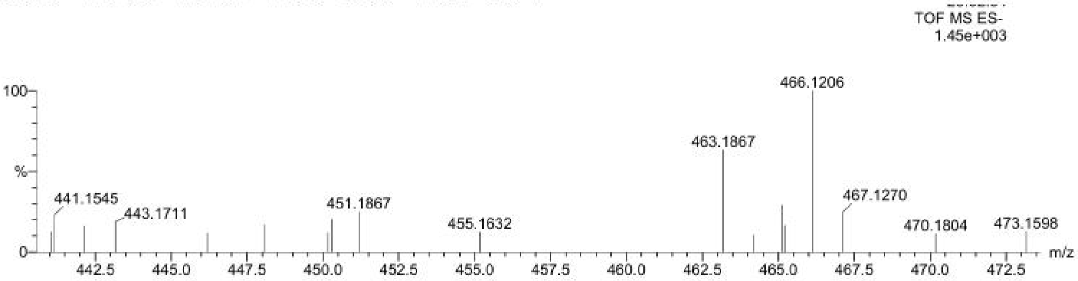

Minimum:				-1.5			
Maximum:		30.0	50.0	100.0			
Mass	Calc. Mass	mDa	PPM	DBE	i-FIT	i-FIT (Norm)	Formula
466.1206	466.1208	-0.2	-0.4	15.5	9.5	0.0	C23 H19 11B N3 O3 F2 S

Elemental Composition Report

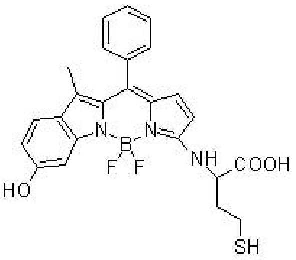

Single Mass Analysis
Tolerance = 30.0 mDa / DBE: min = -1.5, max = 100.0
Element prediction: Off
Number of isotope peaks used for i-FIT = 2

Monoisotopic Mass, Even Electron Ions
279 formula(e) evaluated with 37 results within limits (up to 1 best isotopic matches for each mass)
Elements Used:
C: 0-31 H: 0-33 11B: 0-1 N: 0-3 O: 0-3 F: 0-2 S: 0-1

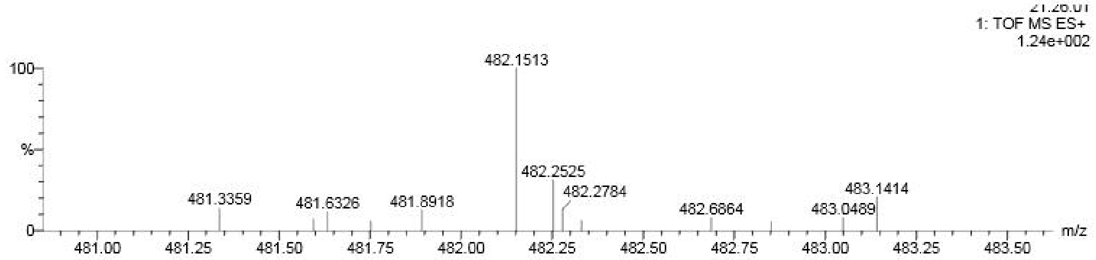

Minimum:				-1.5			
Maximum:		30.0	50.0	100.0			
Mass	Calc. Mass	mDa	PPM	DBE	i-FIT	i-FIT (Norm)	Formula
482.1513	482.1521	-0.8	-1.7	14.5	40.2	0.0	C24 H23 11B N3 O3 F2 S

Fig. S7 HRMS spectra of the products from **S-S-BODIPY-S** with thiols.

9. Time-dependent spectral changes of S-S-BODIPY-S in the presence of Cys/Hcy.

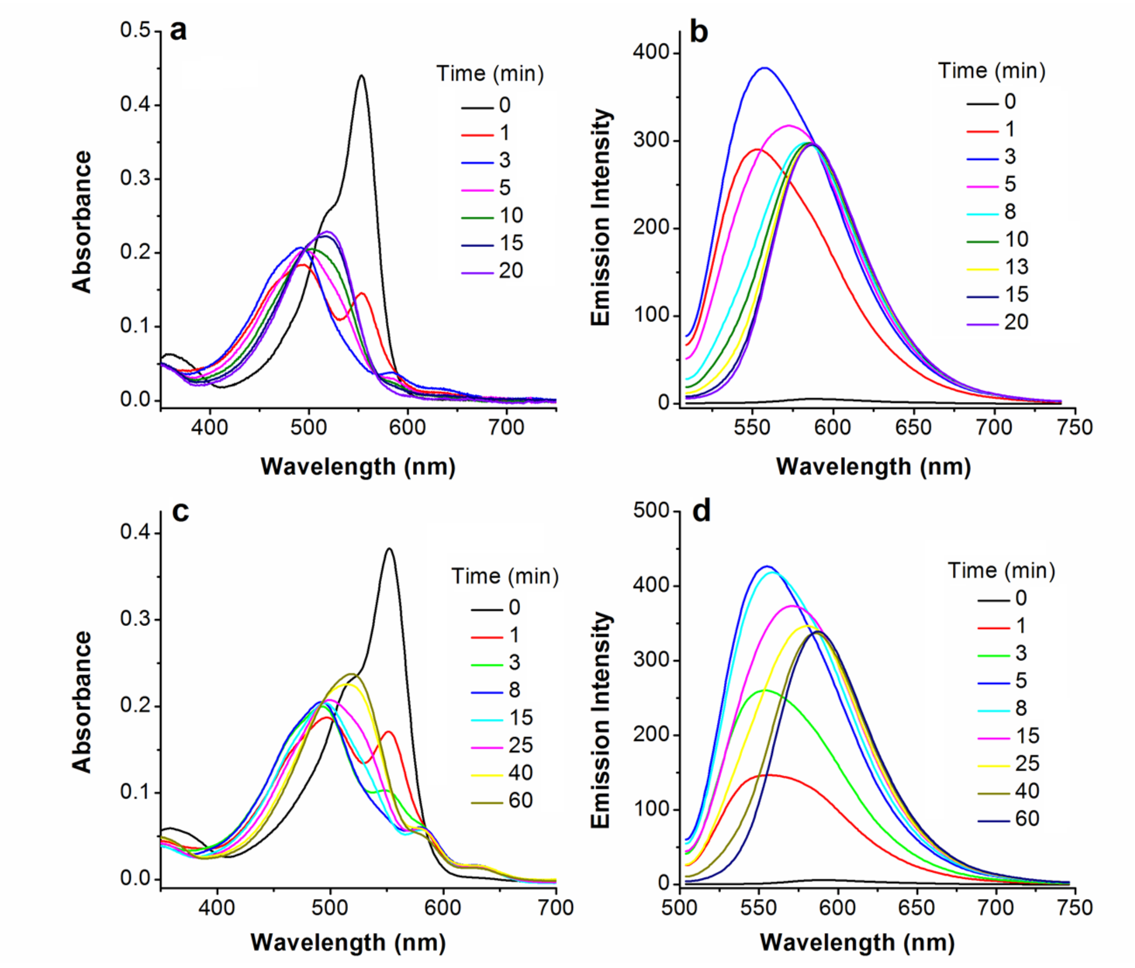

Fig. S8 Time-dependent spectral changes of **S-S-BODIPY-S** (5 μM) in the absence and presence of Cys (5 mM) or Hcy (5 mM) in acetonitrile / HEPES buffer (1:1, v/v , 20 mM, pH 7.4) at 37 °C. Cys: (a) absorption and (b) emission spectra; Hcy: (c) absorption and (d) emission spectra. λ_{ex} = 490 nm.

10. The easy-to-monitor fluorescence color change.

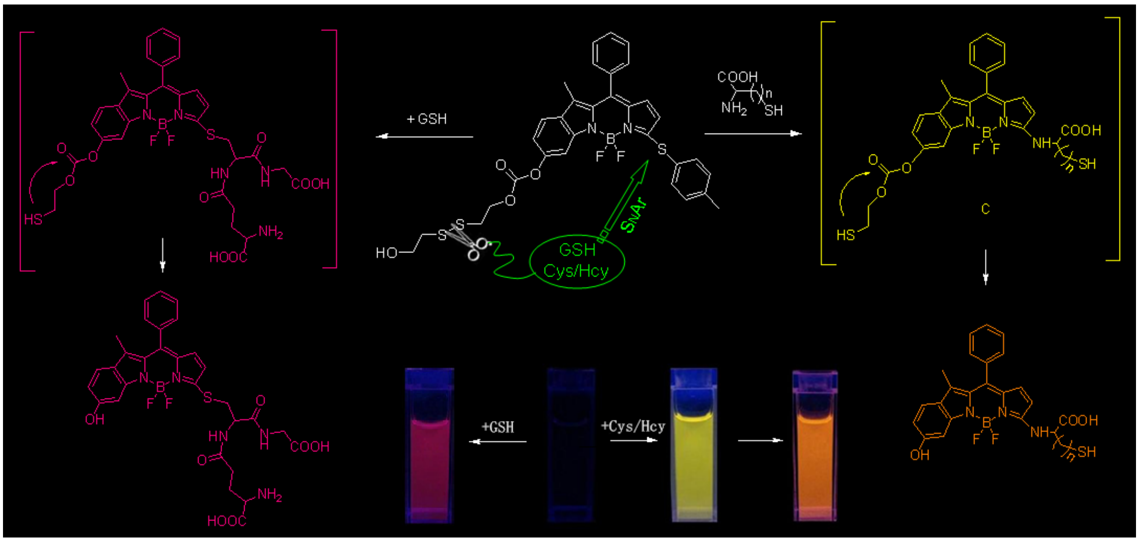

Fig. S9 Fluorescence color changes in the presence of Cys, Hcy and GSH.

11. Fluorescent confocal image of cells.

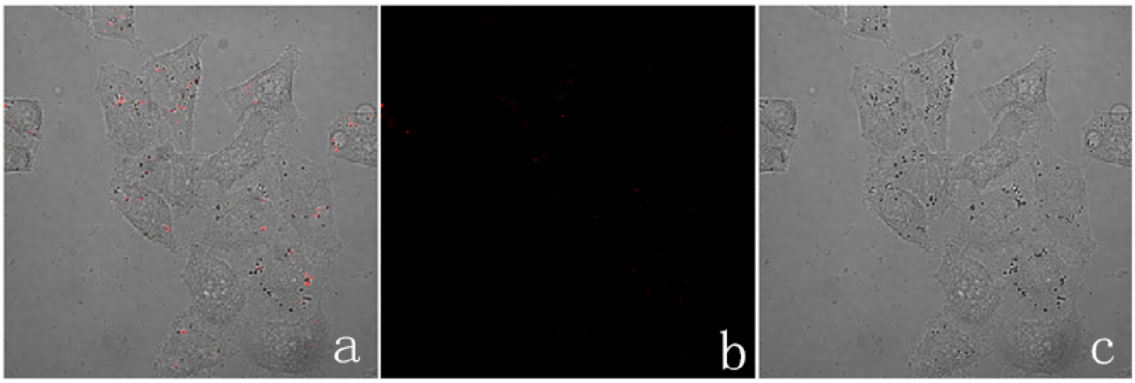

Fig. S10 Fluorescent confocal image of cells: (a-c) HeLa cells pretreated with 500 μM *N*-methylmaleimide for 20 min, then incubated with **S-S-BODIPY-S** (10 μM) for 4 h, the excitation wavelength was 561 nm and the emission was collected at 600-645 nm, (a) overlap field, (b) fluorescence image, (c) bright filed.

12. Experimental section

Materials. Unless other specified, all chemical reagents and solvents for synthesis were purchased from commercial suppliers and were used without further purification. Anhydrous dichloromethane was dried over CaH_2 and distilled prior to use.

Instruments. NMR spectra were recorded on a Bruker AV-400 spectrometer with chemical shifts reported in ppm at room temperature. Mass spectra were obtained on a HP 1100 LC-MS spectrometer. UV-vis absorption spectra were recorded on a Varian Cary 100 spectrophotometer. Fluorescence spectra were measured with a Varian Cary Eclipse Fluorescence spectrophotometer. Spectral-grade solvents were used for measurements of UV-vis absorption and fluorescence. For absorption or fluorescence measurements, compounds were dissolved in CH_3CN to obtain stock solutions (5.0 mM), followed by diluting with aqueous buffer solutions to the desired concentrations.

Cells culture and imaging. HeLa cells and MKN-45 cells were cultured in Roswell Park Memorial Institute 1640 medium (RPMI-1640) supplemented with 10% fetal bovine serum (FBS) in a humidified atmosphere of 5/95 CO_2/air incubator for 24 h at 37 °C. For confocal fluorescence imaging, cells were incubated in glass bottom dishes for 24 h. Cells were incubated at 37 °C with 10 μM **S-BODIPY-S** for 4 h, washed with D-Hanks and fluorescence images were captured. In a control experiment, Hela cells were pre-treated with *N*-methylmaleimide (500 μM) for 20 min at 37 °C, and then washed with D-Hanks. These cells were further loaded with 10 μM **S-BODIPY-S** for 4 h, washed with D-Hanks. Samples were excited at 514 nm and observed between 600-620 nm for the red channel and 560-580 nm for the orange channel.

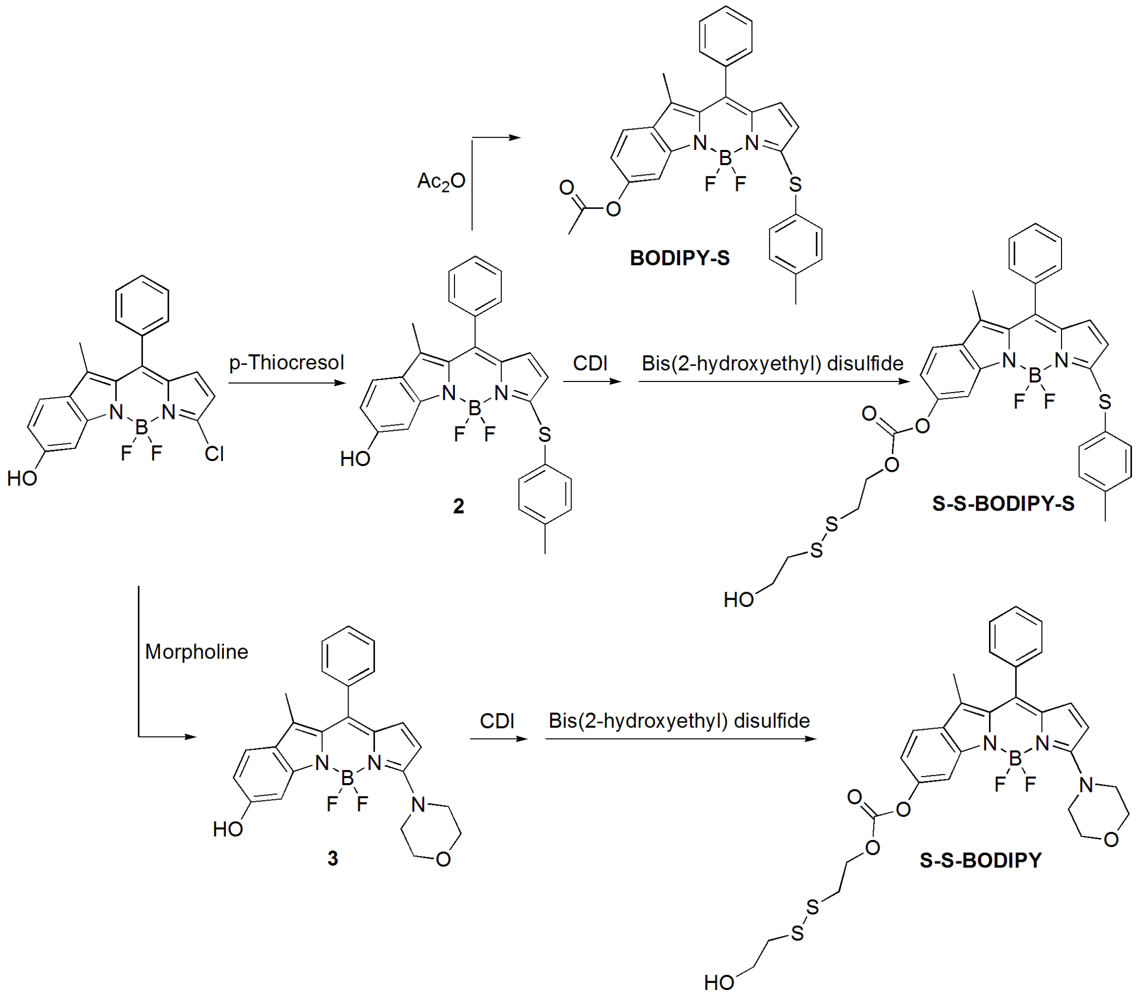

Synthesis of Compound 2. HO-BODIPY-Cl (100 mg, 0.3 mmol) and *p*-thiocresol (39 mg, 0.3 mmol) were dissolved in CH_3CN (30 ml), and then DMAP (38 mg, 0.3 mmol) was added, the mixture was stirred for 1 h at room temperature under N_2 atmosphere. The solvent was then removed under vacuum, the resulting residue was purified by column chromatography on silica gel to afford the compound **2** (102 mg, yield: 83%). ^1H NMR (d_6-DMSO, 400 MHz, ppm): δ 10.51 (s, 1H), 7.60-7.54 (m, 5H), 7.48-7.43 (m, 3H), 7.38-7.36 (d, J = 8.4 Hz, 2H), 6.90 (s, 1H), 6.63-6.60 (dd, J_1 = 2.0 Hz, J_2 = 8.8 Hz, 1H), 6.55-6.53 (d, J = 4.8 Hz, 1H), 5.87-5.86 (d, J = 4.8 Hz, 1H), 2.38 (s, 3H), 1.68 (s, 3H). HRMS (ESI, *m/z*): $[M - H]^-$ calcd for $C_{27}H_{20}BF_2N_2OS$: 469.1357, Found: 469.1364.

Synthesis of Compound 3. To a solution of HO-BODIPY-Cl (0.10 g, 0.3 mmol) in 30 mL CH_3CN was added morpholine (30 mg, 0.3 mmol), the mixture was then stirred for 30 min at room temperature, washed with water, dried over anhydrous Na_2SO_4. The solvent was evaporated in vacuo. The residue was purified by flash chromatography (silica gel, eluent: ethyl acetate/petroleum 1:5) to afford compound **3** (0.100 g, yield: 88%). ^1H NMR (d_6-DMSO, 400 MHz, ppm): δ 9.41 (s, 1H), 7.55-7.54 (m, 3H), 7.40-7.37 (m, 2H), 7.29-7.26 (d, J = 8.8 Hz, 1H), 6.96 (s, 1H), 6.84-6.83 (d, J = 5.2 Hz, 1H), 6.68-6.67 (d, J = 5.2 Hz, 1H), 6.54-6.51 (dd, J_1 = 2.0 Hz, J_2 = 8.8 Hz, 1H), 4.07-4.05 (t, J = 4.4 Hz, 4H), 3.82-3.80 (t, J = 4.4 Hz, 4H), 1.56 (s, 3H). HRMS (ESI, *m/z*): $[M + H]^+$ calcd for $C_{24}H_{23}BF_2N_3O_2$: 434.1851, Found: 434.1846.

Compound BODIPY-S. To a solution of Compound **2** (50 mg, 0.1 mmol) and DMAP (20 mg, 0.2 mmol) in 30 mL anhydrous DCM was added acetic anhydride (16 mg, 0.2 mmol) at room temperature, the reaction mixture was stirred for 1 h under argon. Then the reaction

mixture was washed with water. The organic layer was dried with Na_2SO_4, and evaporated under vacuum. The resulting residue was purified by column chromatography on silica gel to get compound **BODIPY-S** (45 mg, yield 83%). ^1H NMR ($CDCl_3$, 400 MHz, ppm): δ 7.58-7.56 (d, J = 8.4 Hz, 2H), 7.52-7.50 (m, 4H), 7.46-7.44 (d, J = 8.8 Hz, 1H), 7.37-7.35 (m, 2H), 7.29-7.27 (d, J = 8.0 Hz, 2H), 6.81-6.78 (dd, J_1 = 2.0 Hz, J_2 = 8.8 Hz, 1H), 6.58-6.57 (d, J = 4.8 Hz, 1H), 5.94-5.92 (d, J = 4.8 Hz, 1H), 2.42 (s, 3H), 2.33 (s, 3H), 1.74 (s, 3H); ^{13}C NMR ($CDCl_3$, 100 MHz, ppm): δ 169.43, 152.14, 145.89, 141.25, 141.09, 141.02, 135.40, 134.09, 132.35, 132.18, 130.75, 129.64, 129.54, 129.29, 128.60, 124.62,121.91, 121.36, 116.16, 107.49, 29.71, 21.18, 11.85. HRMS (ESI, *m/z*): $[M + Na]^+$ calcd for $C_{29}H_{23}BF_2N_2NaO_2S$: 535.1434, Found: 535.1439.

Compound S-S-BODIPY. Compound **3** (100 mg, 0.2 mmol) was dissolved in 1,2-dichloroethane. 1,1'-Carbonyldiimidazole (75 mg, 0.5 mmol) was added to the above solution, the reaction mixture was stirred for 12 h at 40 ºC under argon. To the reaction mixture was added bis(2-hydroxyethyl)disulfide (106 mg, 0.7 mmol), the resulting reaction mixture was then refluxed for 24 h. After cooling to room temperature, the solvent was removed under vacuum. The resulting residue was purified by column chromatography on silica gel (ethyl acetate / petroleum = 1: 7) to afford the compound **S-S-BODIPY** as a red solid (28 mg, yield 20%). ^1H NMR ($CDCl_3$, 400 MHz, ppm): δ 7.56 (s, 1H), 7.49-7.47 (m, 3H), 7.45-7.42 (d, J = 8.8 Hz, 1H), 7.35-7.33 (m, 2H), 6.87-6.84 (dd, J_1 = 2.0 Hz, J_2 = 8.8 Hz, 1H), 6.75-6.74 (d, J = 5.2 Hz, 1H), 6.34-6.33 (d, J = 5.6 Hz, 1H), 4.54-4.50 (t, J = 6.8 Hz, 2H), 4.08-4.06 (m, 4H), 3.93-3.89 (m, 6H), 3.06-3.03 (t, J = 6.8 Hz, 2H), 2.93-2.90 (t, J = 5.6 Hz, 2H), 1.67 (s, 3H); ^{13}C NMR ($CDCl_3$, 100 MHz, ppm): δ 153.77, 149.53, 141.94, 139.22, 135.90, 135.07, 132.22, 130.02, 129.75, 128.83, 128.40, 122.00, 120.46, 115.12, 113.83, 106.41, 66.87, 66.21, 60.23, 50.82, 41.60, 36.83, 29.71, 10.79. HRMS (ESI, *m/z*): $[M + Na]^+$ calcd for $C_{29}H_{30}BF_2N_3NaO_5S_2$: 636.1580, Found: 636.1589.

Compound S-S-BODIPY-S. The solution of compound **2** (100 mg, 0.2 mmol) and 1,1-carbonyldiimidazole (45 mg, 0.3 mmol) in 1, 2-dichloroethane (30 mL) was stirred for 24 h at 40 ºC under argon. The progress of the reaction was monitored by TLC, when the starting material disappeared and a new intermediate appeared, the bis(2-hydroxyethyl)disulfide (98 mg, 0.6 mmol) was then added to the reaction mixture, the resulting mixture was refluxed for 48 h. After cooling to room temperature, the reaction mixture was diluted with CH_2Cl_2 and washed with water for 3 times, dried over Na_2SO_4, and the solvent was removed under vacuo. The residue was purified by flash chromatography (silica gel, eluent: ethyl acetat / petroleum 1:10) to afford **S-S-BODIPY-S** (20 mg, yield 14%); ^1H NMR ($CDCl_3$, 400 MHz, ppm): δ 7.61 (s, 1H) , 7.58-7.56 (d, J = 8.4 Hz, 2H), 7.52-7.50 (m, 3H), 7.48-7.45 (d, J = 8.8 Hz, 1H), 7.37-7.35 (m, 2H), 7.29-7.27 (d, J = 8.0 Hz, 2H), 8.87-8.85 (dd, J_1 = 2.0 Hz, J_2 = 8.8 Hz, 1H), 6.60-6.58 (d, J = 4.4 Hz, 1H), 5.95-5.94 (d, J = 4.4 Hz, 1H), 4.56-4.53 (t, J = 6.8 Hz, 2H), 3.94-3.91 (t, J = 5.6 Hz, 2H), 3.08-3.05 (t, J = 6.8 Hz, 2H), 2.95-2.92 (t, J = 5.6 Hz, 2H), 2.42 (s, 3H), 1.74 (s, 3H); ^{13}C NMR ($CDCl_3$, 100 MHz, ppm): δ 153.32, 152.10, 145.52, 141.39, 141.17, 140.98, 135.40, 134.01, 132.54, 131.85, 130.78, 129.75, 129.60, 129.28, 128.63, 124.49, 122.08, 121.57, 115.29, 107.09, 66.46, 60.26, 41.59, 36.73, 21.41, 11.84; HRMS (ESI, *m/z*): $[M + Na]^+$ calcd for $C_{32}H_{29}N_2BS_3F_2O_4Na$: 673.1248, Found: 673.1245.

13. NMR and HRMS Spectra

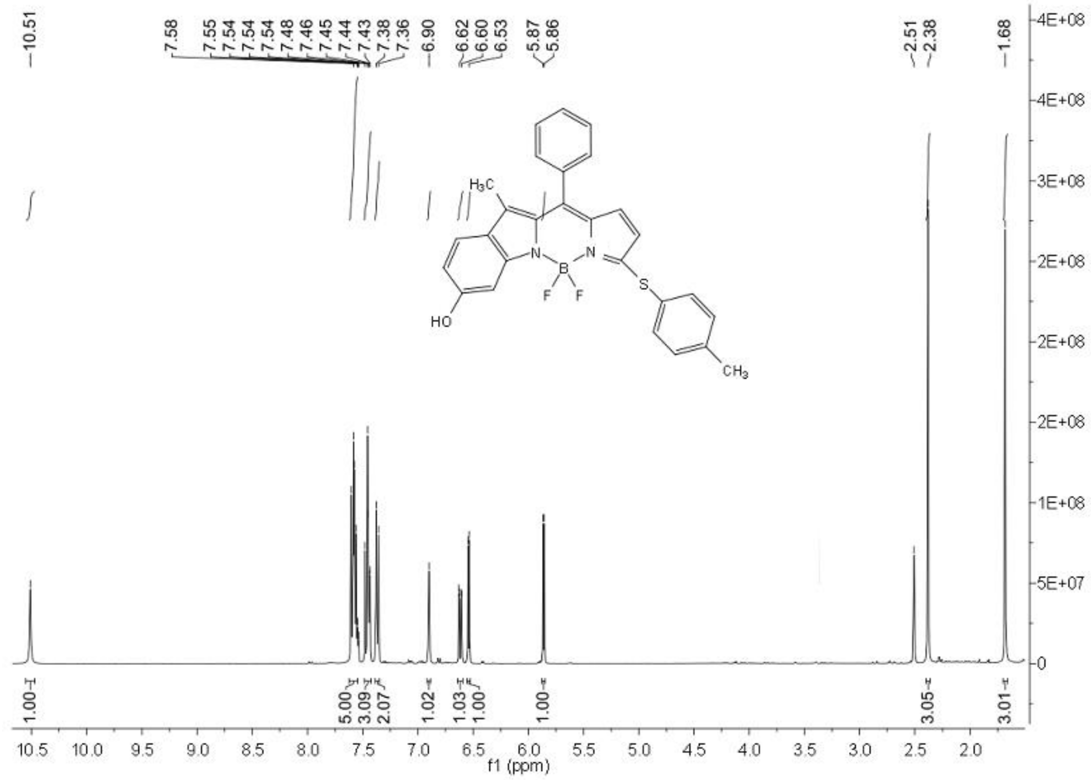

Elemental Composition Report

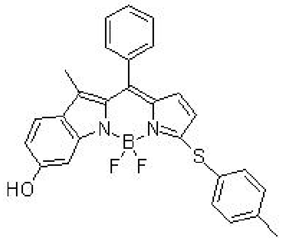

Single Mass Analysis
Tolerance = 30.0 mDa / DBE: min = -1.5, max = 100.0
Element prediction: Off
Number of isotope peaks used for i-FIT = 2

Monoisotopic Mass, Even Electron Ions
125 formula(e) evaluated with 13 results within limits (up to 1 closest results for each mass)
Elements Used:
C: 0-27 H: 0-25 B: 0-1 N: 0-2 O: 0-4 F: 0-2 S: 0-1

ZHAO-CC ECUST institute of Fine Chem 29-Sep-2013 16:21:00

ZCC-WFY-009 78 (0.602) Cm (74:84) 1: TOF MS ES- 1.47e+004

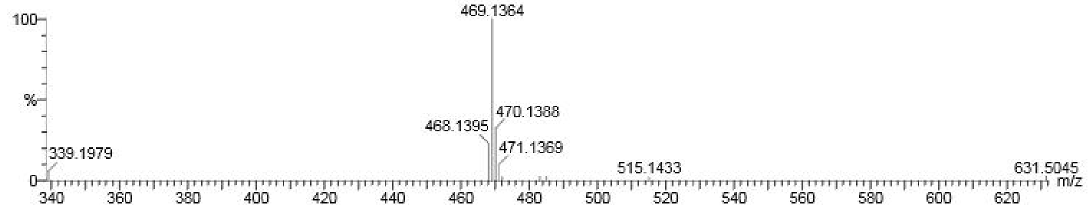

Minimum:				-1.5			
Maximum:		30.0	50.0	100.0			
Mass	Calc. Mass	mDa	PPM	DBE	i-FIT	i-FIT (Norm)	Formula
469.1364	469.1357	0.7	1.5	18.5	24.7	0.0	C27 H20 B N2 O F2 S

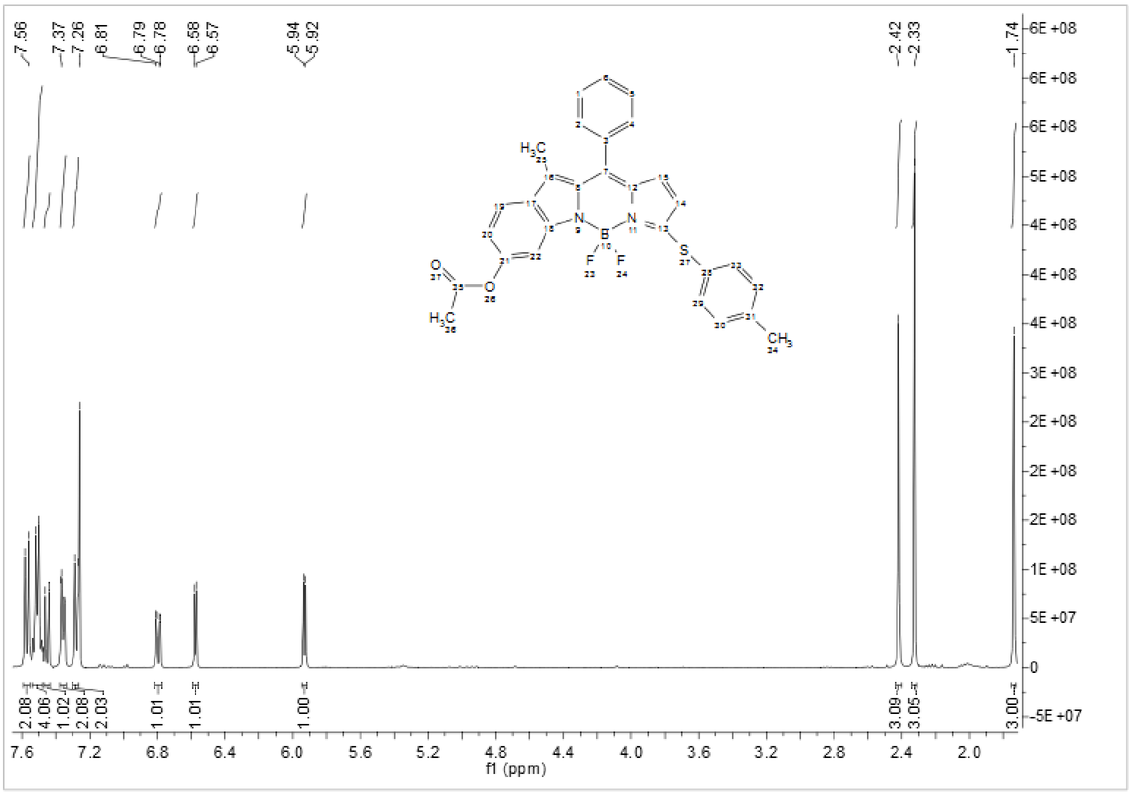

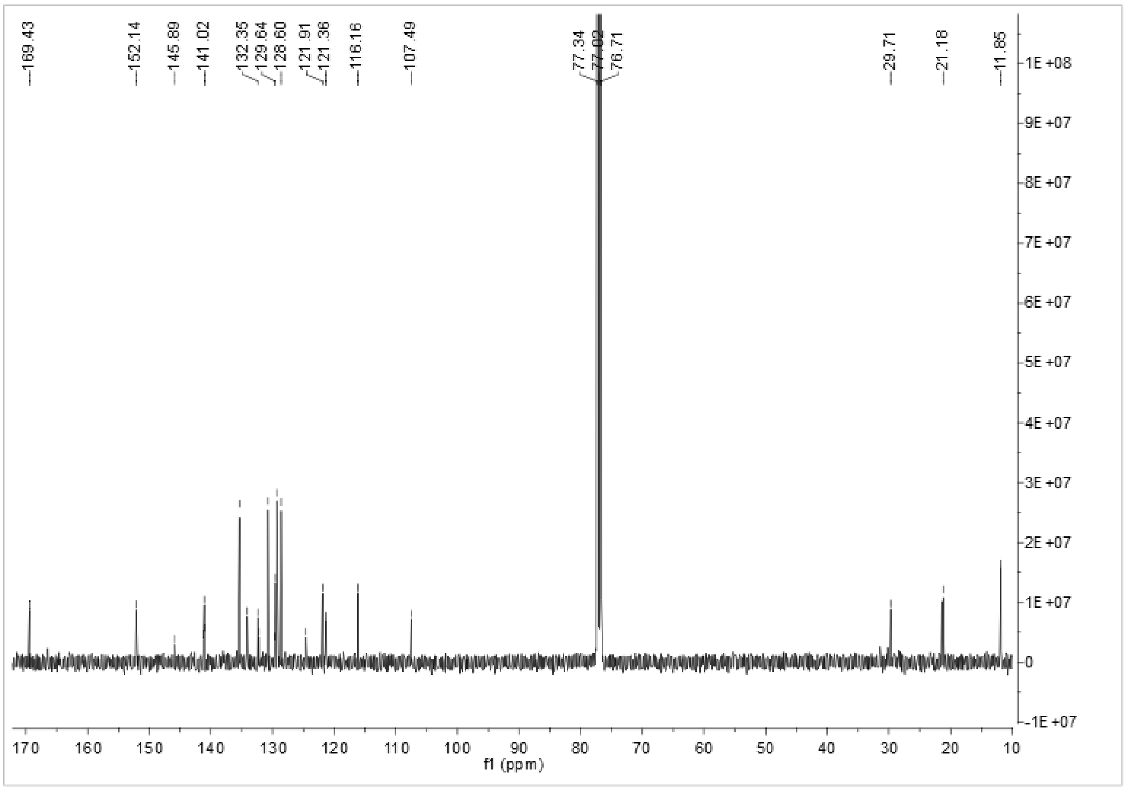

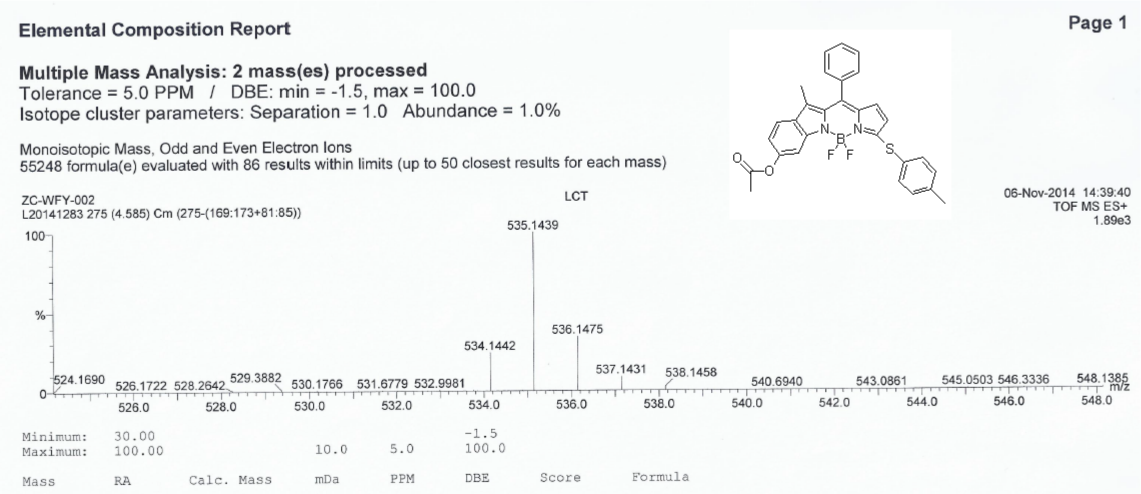

Minimum:	30.00				-1.5		
Maximum:	100.00		10.0	5.0	100.0		
Mass	RA	Calc. Mass	mDa	PPM	DBE	Score	Formula
535.1439	100.00	535.1439	0.0	0.0	18.5	n/a	12C29 H23 B N2 O2 F2 Na S
		535.1440	-0.1	-0.2	31.5	n/a	12C37 13C H17 N3 F
		535.1440	-0.1	-0.2	29.5	n/a	12C37 H20 B N2 S
		535.1441	-0.2	-0.3	24.0	n/a	12C34 13C H21 O2 F2 Na
		[illegible]	[illegible]	[illegible]	[illegible]	[illegible]	12C35 13C H20 B O3 Na

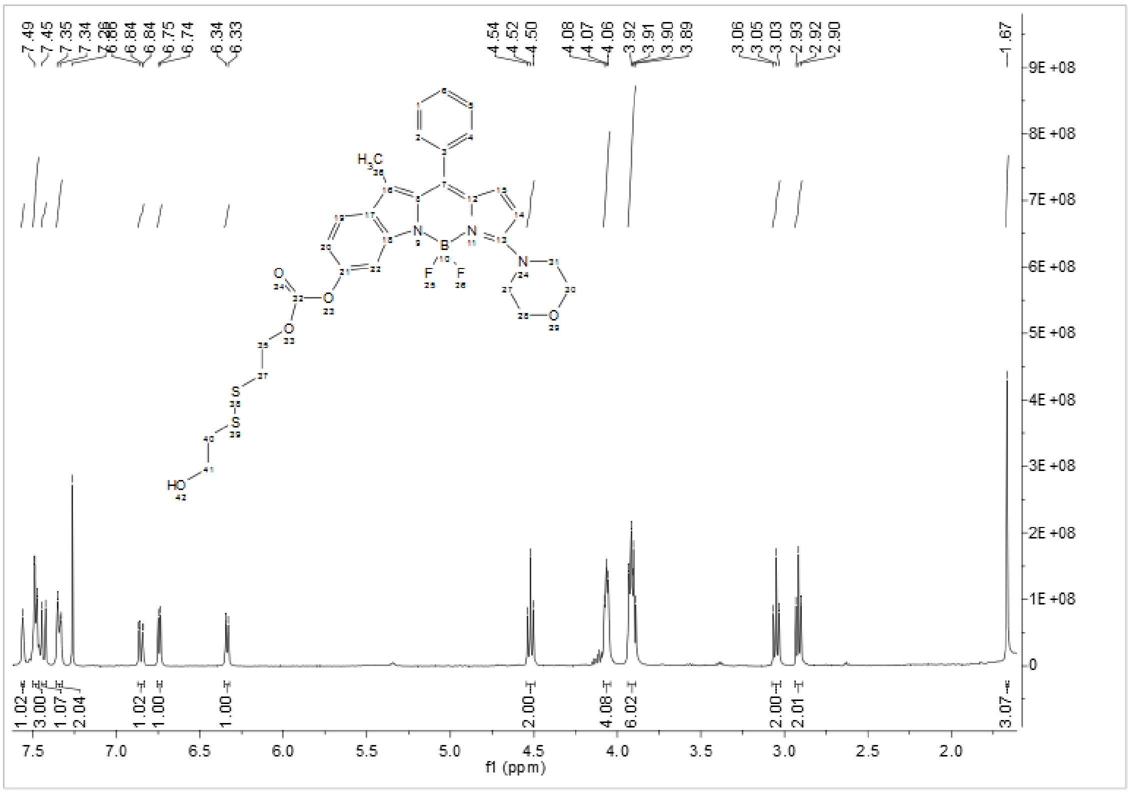

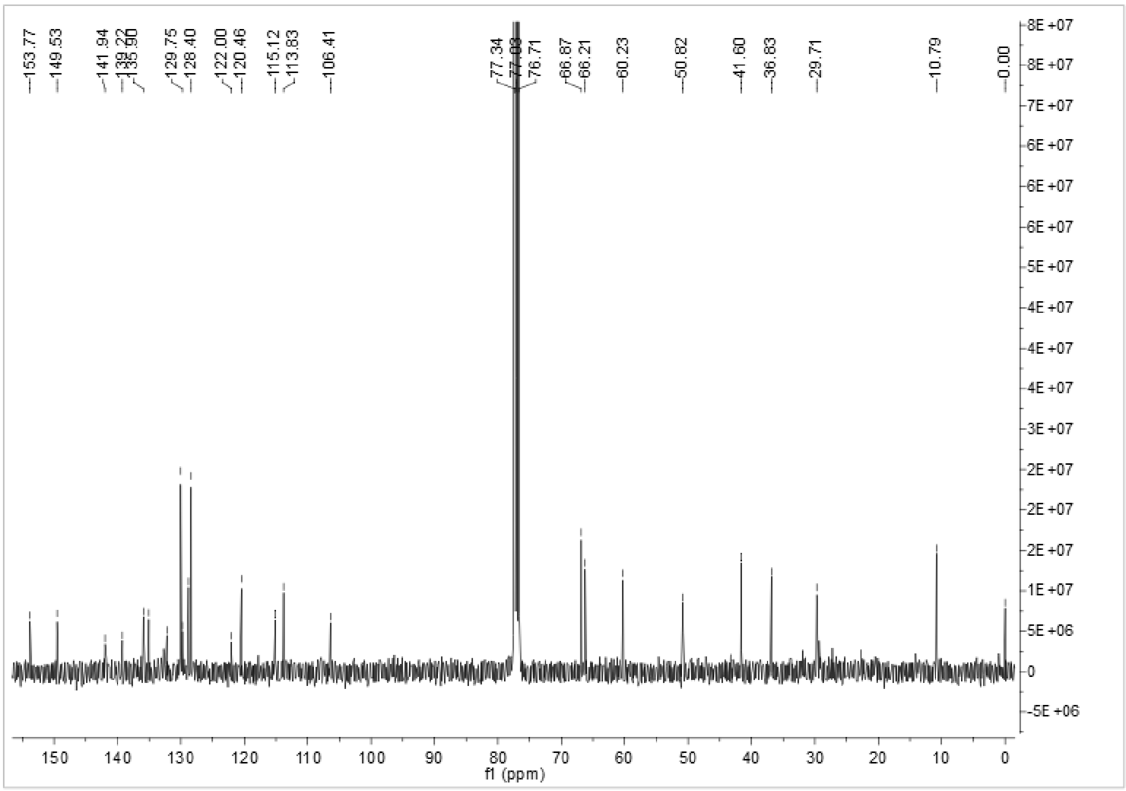

Elemental Composition Report **Page 1**

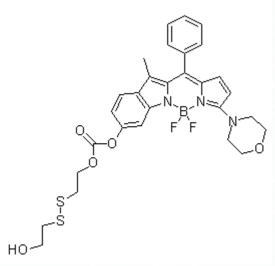

Multiple Mass Analysis: 2 mass(es) processed
Tolerance = 5.0 PPM / DBE: min = -1.5, max = 100.0
Isotope cluster parameters: Separation = 1.0 Abundance = 1.0%

Monoisotopic Mass, Odd and Even Electron Ions
47469 formula(e) evaluated with 89 results within limits (up to 50 closest results for each mass)

ZC-WFY-001 LCT 06-Nov-2014 14:53:54
L20141284 247 (4.118) Cm (247-(156:164+111:117)) TOF MS ES+ 1.19e3

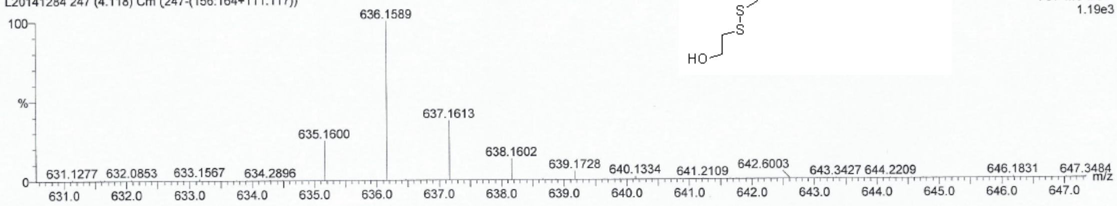

Minimum:	30.00				-1.5		
Maximum:	100.00		10.0	5.0	100.0		
Mass	RA	Calc. Mass	mDa	PPM	DBE	Score	Formula
636.1589	100.00	636.1588	0.1	0.2	29.5	n/a	12C38 13C H22 B N2 O4 F Na
		636.1586	0.3	0.5	15.5	n/a	12C29 H30 B N3 O5 F2 Na S2
		636.1592	-0.3	-0.5	25.0	n/a	12C34 H23 B N4 O6 F Na
		636.1585	0.4	0.6	26.0	n/a	12C36 H23 N4 O4 F2 Na
		636.1593	-0.4	-0.7	27.5	n/a	12C38 H26 N3 O5 S
		[illegible]	[illegible]	[illegible]	[illegible]	[illegible]	[illegible]

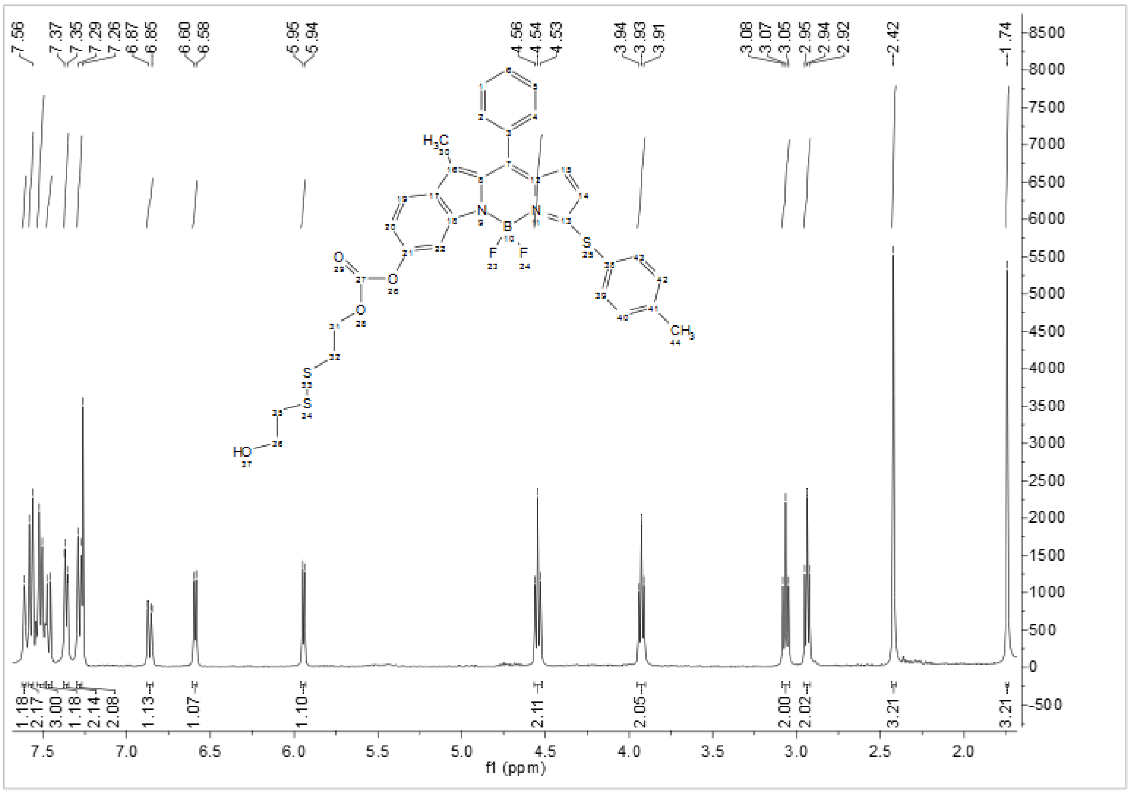

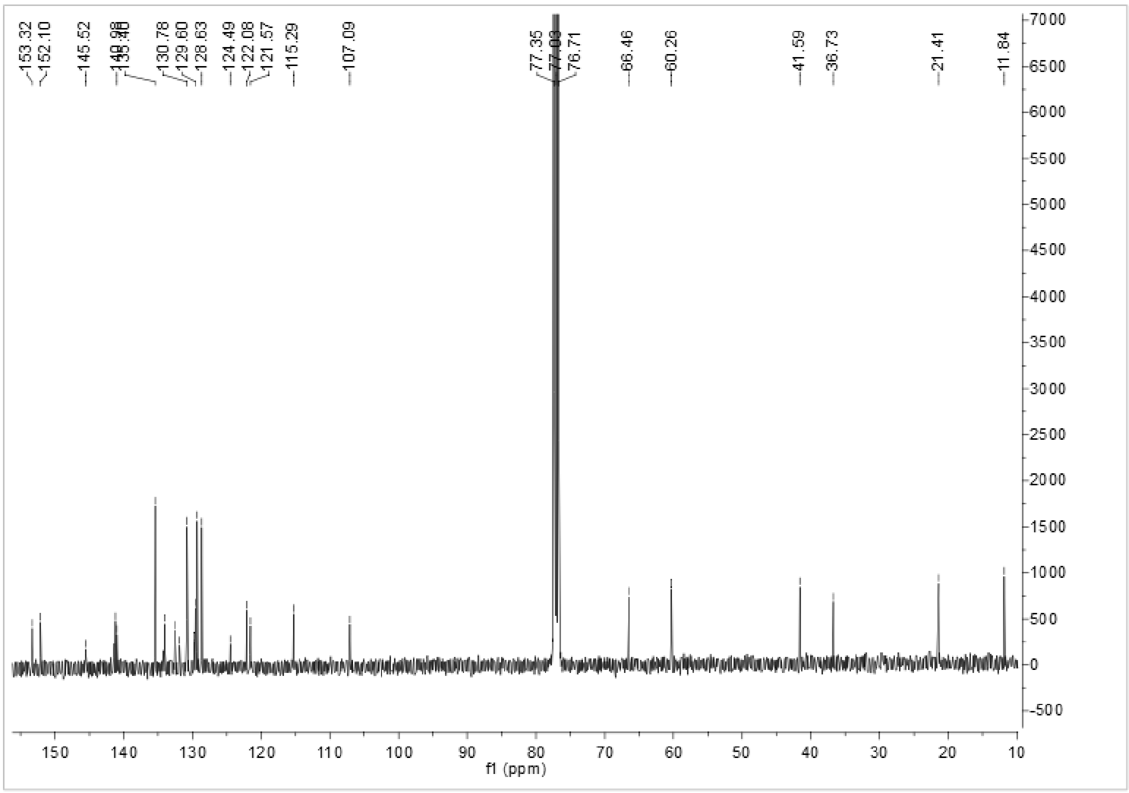

Elemental Composition Report

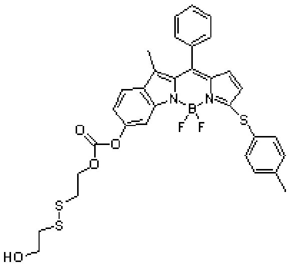

Single Mass Analysis

Tolerance = 100.0 mDa / DBE: min = -1.5, max = 100.0
Element prediction: Off
Number of isotope peaks used for i-FIT = 2

Monoisotopic Mass, Even Electron Ions
740 formula(e) evaluated with 3 results within limits (up to 1 closest results for each mass)
Elements Used:
C: 0-32 H: 0-100 11B: 0-1 N: 0-2 O: 0-4 F: 0-2 Na: 0-1 S: 0-3

CC-ZHAO ECUST institute of Fine Chem 31-Dec-2012 16:04:31

ZCC-WFY-1211 27 (0.914) Cm (21:28) 1: TOF MS ES+ 3.04e+003

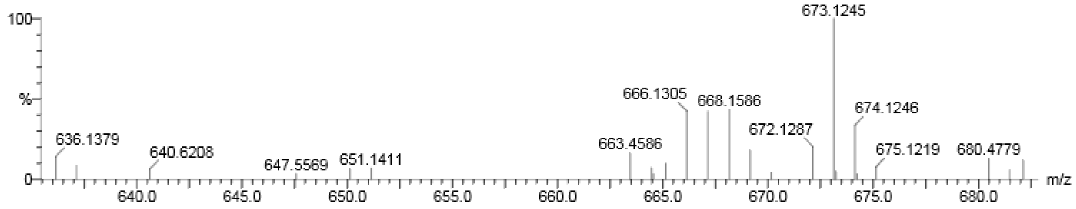

Minimum:				-1.5			
Maximum:		100.0	50.0	100.0			
Mass	Calc. Mass	mDa	PPM	DBE	i-FIT	i-FIT (Norm)	Formula
673.1245	673.1248	-0.3	-0.4	18.5	31.6	0.0	C32 H29 11B N2 O4 F2 Na S3

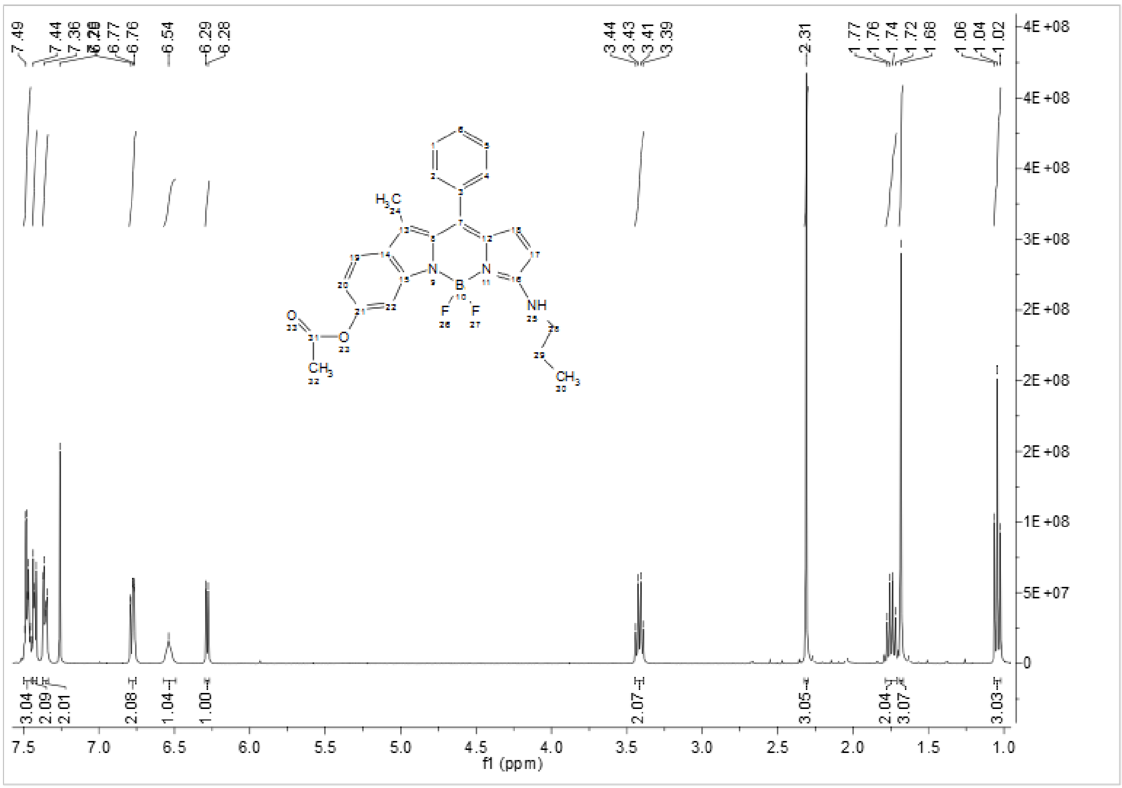

Supplement: Supplementary file 1 [file SC-006-C5SC00216H-s001.pdf]
